# The long-term correlates of developmental stress on whole-brain functional connectivity during emotion regulation

**DOI:** 10.1038/s41398-025-03374-8

**Published:** 2025-04-18

**Authors:** Seda Sacu, Andrea Hermann, Tobias Banaschewski, Martin F. Gerchen, Nathalie E. Holz

**Affiliations:** 1 Department of Child and Adolescent Psychiatry and Psychotherapy, Central Institute of Mental Health, Medical Faculty Mannheim, University of Heidelberg, Mannheim, Germany; 2 German Center for Mental Health (DZPG), partner site Mannheim-Heidelberg-Ulm, Mannheim, Germany; 3 Department of Psychotherapy and Systems Neuroscience, Justus Liebig University, Giessen, Germany; 4 Bender Institute of Neuroimaging, Justus Liebig University, Giessen, Germany; 5 Center for Mind, Brain and Behavior, Phillips University Marburg and Justus Liebig University, Giessen, Germany; 6 Department of Clinical Psychology, Central Institute of Mental Health, Medical Faculty Mannheim, University of Heidelberg, Mannheim, Germany; 7 Department of Psychology, University of Heidelberg, Heidelberg, Germany

**Keywords:** Psychiatric disorders, Predictive markers

## Abstract

Early life stress is associated with alterations in brain function and connectivity during affective processing, especially in the fronto-limbic pathway. However, most of the previous studies were limited to a small set of priori-selected regions and did not address the impact of stress timing on functional connectivity. Using data from a longitudinal birth cohort study (*n* = 161, 87 females, mean age (SD) = 32.2(0.3)), we investigated the associations between different time points of stress exposure and functional connectivity. We measured stressful life events across development using a modified version of Munich Event List and grouped into four developmental stages: prenatal/newborn (prenatal-3 months), infancy and toddlerhood (3 months-4.5 years), childhood (4.5–11 years), and adolescence (11–19 years). All participants completed an fMRI-based emotion regulation task at the age of 33 years. Task-dependent directed functional connectivity was calculated using whole-brain generalized psychophysiological interactions. The association between life stress and connectivity was investigated within a multiple regression framework. Our findings revealed distinct associations between stress exposure and task-specific functional connectivity, depending on the developmental timing of stress exposure. While prenatal and childhood stress were associated with lower connectivity between subcortex and cognitive networks, stress exposure unique to adolescence was related to higher connectivity from the salience network to the cognitive networks. These results suggest that early life stress alters the connectivity of cognitive and limbic networks, which are important for emotion processing and regulation. Future research should replicate and extend the findings regarding sensitive periods by utilizing diverse paradigms in cognitive, social, and emotional domains.

## Introduction

Early life stress (ELS) alters brain development [1] and increases the risk of developing psychopathology later in life [2]. Individuals exposed to ELS often present with emotion regulation difficulties at behavioral [3] as well as neural [4] levels. At the neural level, emotion regulation requires the recruitment of cognitive control regions, such as the prefrontal cortex, and modulation of amygdala activity [5, 6]. Several previous studies reported altered activity and connectivity of limbic and cognitive control regions in individuals exposed to ELS [7]. Healthy adults exposed to ELS showed enhanced amygdala activation in response to negative emotional stimuli [8–10] and disrupted fronto-limbic connectivity during emotion regulation [11, 12]. However, these studies were limited to a small set of priori-selected regions and did not address the impact of stress timing on brain responses during emotion processing.

Most of the previous studies investigated alterations only in the fronto-limbic pathway [13]. Although the fronto-limbic pathway plays a vital role in emotion regulation, and altered fronto-limbic connectivity has been identified in ELS and stress-related psychopathologies [4], less is known about global or whole-brain connectivity alterations following ELS [14]. Previous research suggests the involvement of several large-scale brain networks in emotion processing and regulation beyond the fronto-limbic pathway, such as salience network (attention allocation, implicit emotion regulation), executive control networks (emotion regulation, goal-directed behavior) and default-mode network (mentalizing, autobiographical memory) [15, 16]. Thus, investigating whole-brain connectivity via large-scale brain networks rather than a small set of regions of interest can bring new insights into the neural embedding of ELS.

Most evidence for disrupted large-scale network connectivity following ELS comes from the resting-state fMRI literature [14]. Several studies have reported altered resting-state functional connectivity of large-scale brain networks in individuals exposed to adversity [17–22]. Despite the growing literature on resting-state large-scale network alterations, only one previous study investigated whole-brain connectivity using an affective paradigm [23]. Their findings showed that early life trauma in adolescent girls was associated with more modular but less globally efficient connectivity during the processing of fearful and neutral faces, irrespective of emotional valance. While these findings provided compelling evidence for global measures of connectivity in adolescents, it remains unclear how ELS is associated with alterations in large-scale brain networks during affective processing in healthy adults.

Recently, growing interest has emerged in identifying sensitive periods for stress exposure—time windows of increased vulnerability to stress [24–26]. A sensitive period suggests that stress occurring at different life stages might have distinct effects on a neural system of interest [27]. In line with this, a recent study showed that abuse during childhood, but not during adolescence, was related to intrinsic functional connectivity alterations in several large-scale networks [28]. Their findings underscored the importance of the developmental timing of stress (childhood versus adolescence stress) in intrinsic adult brain connectivity. However, studies examining the long-term impact of stressors occurring very early in life (i.e., under age 3) remain scarce. Although several previous studies have examined the impact of early life stressors on infant brain connectivity [29–32], only one recent study investigated the long-term effects of a very early life stressor (e.g., prenatal maternal anxiety) in adults [33]. However, to the best of our knowledge, no previous study has examined the impact of lifespan stress (i.e., stress occurring at different developmental stages across a broad temporal spectrum) on adult brain connectivity.

Previous literature used diverse ELS measures, including major adverse life events [17], childhood trauma [18–20], institutionalization [21], and prenatal maternal psychopathology symptoms [31–33]. Most of these stressors reflected relatively rare events or severe adversities. It is, therefore, less clear how general stress exposure can affect functional network organization in adults. Cumulative risk exposure has a profound effect on mental health [34, 35] and brain alterations [36, 37] and offers a greater sensitivity to identifying the effect of varying levels of stress [38]. Hence, investigating the cumulative effect of life stressors on functional connectivity will bring additional insights into the existing literature.

The current study aimed to investigate the association between life stress and directed functional connectivity during an emotion regulation task in healthy adults. All participants underwent a task-based fMRI and completed psychopathology measures at the age of 33 years. Directed connectivity, defined as the influence one brain region exerts over another [39], was estimated using whole-brain generalized psychophysiological interactions [40]. Examining directed connectivity provides insights into the information flow between brain regions [41], which is crucial for understanding the specific pathways through which life stress impacts emotion regulation processes. In addition, by estimating the functional connectivity during an affective paradigm, we had greater control over the measured cognitive state and investigated a specific behavioral domain (i.e., emotion regulation), which was consistently found to be affected by ELS exposure [42].

In this study, we focused on exposure to stressful events before adulthood. For this purpose, we used a prospectively collected stress measure that covered different stages of life, including prenatal, infancy, childhood, and adolescence periods. By using a stress measure spanning a large temporal spectrum (prenatal to 19 years), we were able to examine the distinct effect of stress exposure on functional connectivity across development. We hypothesized that ELS would be associated with alterations in emotion and attention networks. Specifically, ELS would be associated with alterations in salience, limbic and frontoparietal network connectivity during emotion regulation, given the importance of these networks in emotion regulation [15, 16] and the functional abnormalities identified in these networks following ELS [21]. We did not put forward a specific hypothesis regarding the direction of changes due to scarce evidence. Additionally, we expected that these network alterations would be linked to psychopathology symptoms.

## Materials and methods

### Participants

The present study was conducted within the framework of the Mannheim Study of Children at Risk, a longitudinal birth cohort study designed to investigate long-term outcomes of early psychosocial and biological risk factors on development [43]. The initial sample included 384 children born between 1986 and 1988. The participants were followed from their birth up to the age of 33 years across 11 assessment waves (Supplementary Fig. S1). At the last assessment wave (T11), 170 participants had functional MRI data available. Two participants were excluded due to inefficient coverage of the brain surface during the task. Additional seven participants were excluded due to excessive head motion (>3 mm in transition or 3 degrees in rotation). The final sample included 161 participants (mean age = 32.21 years, 87 females). At the time of fMRI assessment, 21 (13%) participants had current psychopathology, which was confirmed by German version of the Structured Clinical Interview for DSM-IV [44] (see Table 1 for Sample Characteristics).

**Table 1:** Sample characteristics.

*N* = 161	
Age, M (SD)	32.21 (0.29)
Sex, N (%), Female	87 (54%)
Current psychopathology, N (%)	21 (13%)
Major Depressive Disorder	7 (4.3%)
Anxiety Disorder	8 (5%)
Alcohol and Substance Abuse	4 (2.5%)
Attention Deficit and Hyperactivity Disorder	1 (<1%)
Schizophrenia	1 (<1%)
Internalizing problems (T11), Median (IQR)	7 (12)
Externalizing problems (T11), Median (IQR)	5 (8)

The study was approved by the ethics committee of the University of Heidelberg. All participants gave informed consent. All methods were performed in accordance with relevant guidelines and regulations, including the Declaration of Helsinki.

### Psychological measurements

#### Stressful life events

Stressful life events were measured from the first assessment wave (age of 3 months) until the last assessment wave (age of 33 years) using a modified version of the Munich Event List [45]. The items of the life stress questionnaire are specifically designed to cover positive and negative stressors relevant to various aspect of development, addressing domains such as partnership, education, work, health, and finance. The events included both normative (i.e., expected life changes, such as school and job transitions, marriage, childbirth, moving) and non-normative (i.e., unexpected life changes, such as loss of loved ones, separation, hospitalization, incarceration) stressors [46]. While normative changes are generally anticipated, they can also be stressful, particularly when accompanied by additional demands or challenging circumstances. In addition, stressors differ in terms of duration (acute vs. chronic events (>3 months)). Between T1 and T6 (15 years), trained psychologists conducted a standardized interview with caregivers. At T7 (19 years), participants rated life events themselves.

We here measured exposure to stressful life events based on presence of a stressful event (i.e., stimulus-based stress approach), independent from their valance, chronicity and magnitude. For each time point, we first calculated total scores based on the frequency of event occurrence reflecting cumulative risk [38]. Since the item numbers differed across assessment waves (Table S1), the total scores were standardized using z-transformation. Importantly, the vast majority of our participants had stress exposure but the amount of stress exposure varied significantly across participants (Table S1, Fig. S2). To represent developmental stages, we used the following sum scores: prenatal period and newborn (from pregnancy to up to postnatal 3 months), infancy and toddlerhood (three months to 4.5 years), childhood (4.5 years to 11 years), and adolescence (11 years to 19 years) [47]. Here, we are interested in ELS (i.e., stress occurring prior to adulthood) and used the data through adolescence. The sum scores of life events that occurred in the last 12 months prior to the fMRI measurement was used to control the effect of current life stress on the main analysis. Life events variables showed small-to-moderate correlations between each other and did not correlate with current life stress (Supplementary Table S2).

#### Psychopathology

The Adult Self-Report [48] was used to assess current symptoms of psychopathology at the time of the fMRI assessment (age 33 years), which includes 126 items rated on a 3-point Likert scale (0= ‘not true’, 1= ‘somewhat or sometimes true’, 2= ‘very true or often true’). For the current study, we used internalizing and externalizing problems scores only.

#### Emotion regulation strategies

We measured habitual use of two common emotion regulation strategies (i.e., reappraisal and suppression) using the German version of Emotion Regulation Questionnaire [49].

### Experimental paradigm

We used a modified version of the block-designed emotion regulation task [50] to examine neural correlates of emotion regulation. The task consisted of three experimental conditions: Look neutral, look negative and regulate (Supplementary Fig. S3). During the look neutral blocks, participants were asked to simply look at neutral images. During the look negative blocks, participants were asked to attend to negative images without trying to change or alter the emotional state elicited by the images. In the regulate blocks, participants were instructed to decrease their negative affect elicited by the negative image using one of two reappraisal strategies (i.e., distancing and rationalizing). Stimuli were selected from the International Affective Picture System [51] and the internet [50] and validated based on valence and arousal ratings [50], proving that negative images were perceived less pleasant and more arousing. The pictures displayed scenes in which at least one person depicted in an aversive (e.g., homeless person, accidents, ill person at a hospital) or neutral (e.g., two people in a conservation) context. Immediately following the experimental block, participants were asked to rate the intensity of their negative affect on a Likert-type 7-point scale. The total task comprised random presentation of four blocks of each condition and lasted for 6 min 37 s.

### MRI data acquisition and preprocessing

The functional and structural images were acquired on a Siemens Magnetom Prisma Fit (Siemens, Erlangen, Germany) 3 T MRI scanner with a standard 32-channel head coil. During the emotion regulation task, 186 volumes were obtained using a gradient echo-planar sequence sensitive to blood oxygen level-dependent (BOLD) contrast (36 slices, TE = 35 ms, TR = 2100 ms, voxel size = 3 × 3 × 3 mm). The first 11 scans were discarded to allow for equilibration of the magnetic field. Preprocessing was performed using SPM 12 and included the following steps: slice-timing correction, realignment, structural and functional image co-registration, segmentation, normalization to the Montreal Neurological Institute (MNI) 152 template, and smoothing using a kernel with a FWHM of 8 mm. Frame-wise displacement (M = 0.14, SD = 0.06) was calculated based on six motion parameters to quantify head motion [52].

### First level analyses

#### Generalized-linear modelling

Experimental conditions were convolved with a canonical hemodynamic response function using generalized linear modeling implemented in SPM 12. Six motion parameters and time series from white matter and cerebrospinal fluid were entered into the subject-level analysis as nuisance covariates to correct for motion and physiological noise. Additionally, we censored the scans (as well as preceding and following scans) with frame-wise displacement greater than 0.5 mm [23].

#### Brain parcellation

Brainnetome atlas [53] was used to identify brain regions for functional connectivity analysis. In total, the atlas included 246 regions (210 cortical and 36 subcortical regions). Time series were extracted using the first eigenvariate of all voxels within the regional masks using SPM.

#### Functional connectivity analysis

We used whole-brain psychophysiological interactions (PPI) [40] to estimate directed functional connectivity during the emotion regulation task. PPIs explain regional responses of one brain area (target region) in terms of an interaction between the activity of another brain area (see region) and the time course of experimental conditions (task-related changes). Similar to conventional PPI approaches [39, 54], whole-brain PPI uses a regression model including the psychological term (experimental condition), the physiological term (regional time series of a seed region), interactions between psychological and physiological terms and covariates of no-interests. In line with a previous suggestion [55], whole-brain PPI centers the psychological variable to prevent spurious PPI effects. Different from conventional PPI approaches, the analysis is conducted in a whole-brain manner using priori-selected regions, however, not all regions included in the analysis show condition specific activation as task-dependent connectivity can change in the absence of condition-specific activation [56].

In addition, whole-brain PPI provides information about directionality (i.e., directed functional connectivity). In the whole-PPI approach, a functional coupling metric includes seed (e.g., a region sending information) and target (e.g., a region receiving information) regions. Since the method is based on a GLM framework, changing the position of seed and target regions in the equation will result in different connectivity estimations. Therefore, each connection will have a unique t-value estimation, making the connectivity matrix non-symmetric. Whole-brain PPI analysis was performed in MATLAB software (2024a) using in-house script utilizing SPM12 functions. The first level analysis included regional time-series from 246 brain regions as input. A whole-brain (246 × 246) connectivity matrix was estimated for each experimental condition separately. Each connection metric had a t-value estimation (and corresponding p-value) representing the connection strength. The emotion regulation contrast (regulate negative – look negative) was calculated by subtracting the connectivity matrix for the look negative condition from the matrix for the regulate negative condition, to obtain the connectivity estimates specific to emotion regulation. While we used whole-brain PPI to estimate task-dependent connectivity changes, on a conceptual level this is similar to investigating differences of condition-specific Pearson’s correlations, which are, however, based on a simpler model and require additional assumptions about how time points are assigned to conditions.

### Second-level analyses

#### Brain activation

A one-sample t-test was performed using the emotion regulation (regulate > look negative) contrast to identify brain regions showing task effect (*p* < 0.05, whole-brain family-wise error (FWE) corrected). We also performed multiple regression analysis to investigate associations between stress exposure and task-related activity during emotion regulation (*p* < 0.05, cluster-level FWE corrected).

#### Functional connectivity

We performed multiple regression analysis using Network-based statistics (NBS) [57, 58] to find associations between ELS and task-dependent connectivity. In total, we performed four regression analyses, each including the stress measure from a different developmental period as the covariate of interest and controlled for sex, current psychopathology, current life stress, and mean framewise displacement. To account for the specific effects of each developmental period, we conducted additional regression models that included stress from other developmental periods as covariates of no interest in addition to the base covariates mentioned above.

A conservative t-statistic threshold (t > 3.5 for positive connections/associations and t < −3.5 for negative connections/associations) [19] was chosen to identify the connections forming a network component (i.e., connected edges). These network components were then corrected for multiple comparisons by estimating the likelihood of clusters with similar sizes occurring by chance through permutation testing (5000 iterations). This procedure corrects network components rather than single connections, which is akin to cluster-level FWE correction in classical fMRI analysis. In addition, we calculated effect sizes for each connection using Hedge’s g, the bias-corrected version of Cohen’s d [59].

### Brain- behavior relationship

Having determined the connections associated with developmental stress, we extracted connectivity estimates (i.e., regression coefficients) of those connections from subject-level connectivity matrices. The associations between the connectivity values and psychopathology measures were then examined within the regression framework using ordinary least squares approach. A false discovery rate (FDR) correction was applied to the associations within each developmental period.

### Statistical analysis

Statistical analyses were conducted using IBM SPSS, Version 27. A t-test for dependent samples was performed to see if emotion regulation is successful. Additionally, we conducted several correlation analyses to examine associations between life stress, psychopathology, and regulation success, and habitual use of emotion regulation strategies. Spearman’s rank-order correlation test was performed when the assumption are not met. The behavioral results were corrected for multiple testing (i.e., four developmental stages) using FDR correction.

### Sensitivity analysis

#### Brain parcellation

Selection of a brain parcellation map is a subjective process, which could introduce heterogeneity [60, 61]. To reduce this bias in our connectivity analyses, we repeated our analyses using two other commonly used brain atlases: Automated Anatomical Labelling [62] representing anatomical parcellation and Schaefer atlas [63] representing functional parcellation with similar features (e.g., region number, network assignment).

#### Impact of self-report

Stress exposure scores were based on the parental reports up to 15 years (T6). Although youth reported life events at the age of 15 via the short-version of event list, we opted to use the parent version to keep the number of item compatible with the 19-years measurement, which was based on self-report. Consequently, adolescent stress scores reflected information from different sources. To ensure that results were not affected by information source, we conducted a sensitivity analysis by calculating adolescent stress scores solely based on self-report.

#### Regulation strategy

Participants were asked about their emotion regulation strategy after the completion of the fMRI task. Most of the participants (*n* = 129) used reappraisal to regulate their emotions, while 32 of them failed to use any strategy. We repeated the main analyses by excluding those participants who did not use reappraisal to regulate their emotions.

### Stress and habitual use of emotion regulation strategies

We additionally checked whether habitual use of emotion regulation strategies is related to stress timing, psychopathology, and regulation success during the task.

## Results

### Behavioral results

Participants rated the intensity of their negative affect higher in the look negative condition (M = 4.76, SD = 1.28) than in the emotion regulation condition (M = 3.38, SD = 1.24; *t* (160) = 13.91, *p* < 0.001), showing that emotion regulation was successful at the behavioral level. We also calculated emotion regulation success based on rating score (look negative – regulate negative). Life stress during adolescence was negatively associated with emotion regulation success (r = −0.23, FDR-*p* = 0.012). Life stress during childhood (rs = 0.23, FDR-*p* = 0.012) and adolescence (rs = 0.19, FDR-*p* = 0.038) were associated with higher internalizing symptoms in adulthood. Similarly, life stress during childhood (rs = 0.19, FDR-*p* = 0.028) and adolescence (rs = 0.21, FDR-*p* = 0.028) were associated with higher externalizing symptoms in adulthood.

### Task-related brain activation

During emotion regulation (regulate > look negative), there was increased activation in several brain regions including frontal, parietal, and temporal cortices and cerebellum, whereas insula, superior temporal gyrus and right precentral gyrus showed decreased activation (*p* < 0.05, whole-brain FWE-corrected; Fig. S4).

### Developmental stress and task-related brain activation

Stress during childhood was related to higher activation in the left fusiform gyrus and cerebellum (k = 83, t = 4.52, *p* < 0.05 cluster-level FWE-corrected) during emotion regulation (Fig. S5). No other stress measure was significantly associated with the brain activation.

### Task-dependent connectivity

**Fig. 1:**
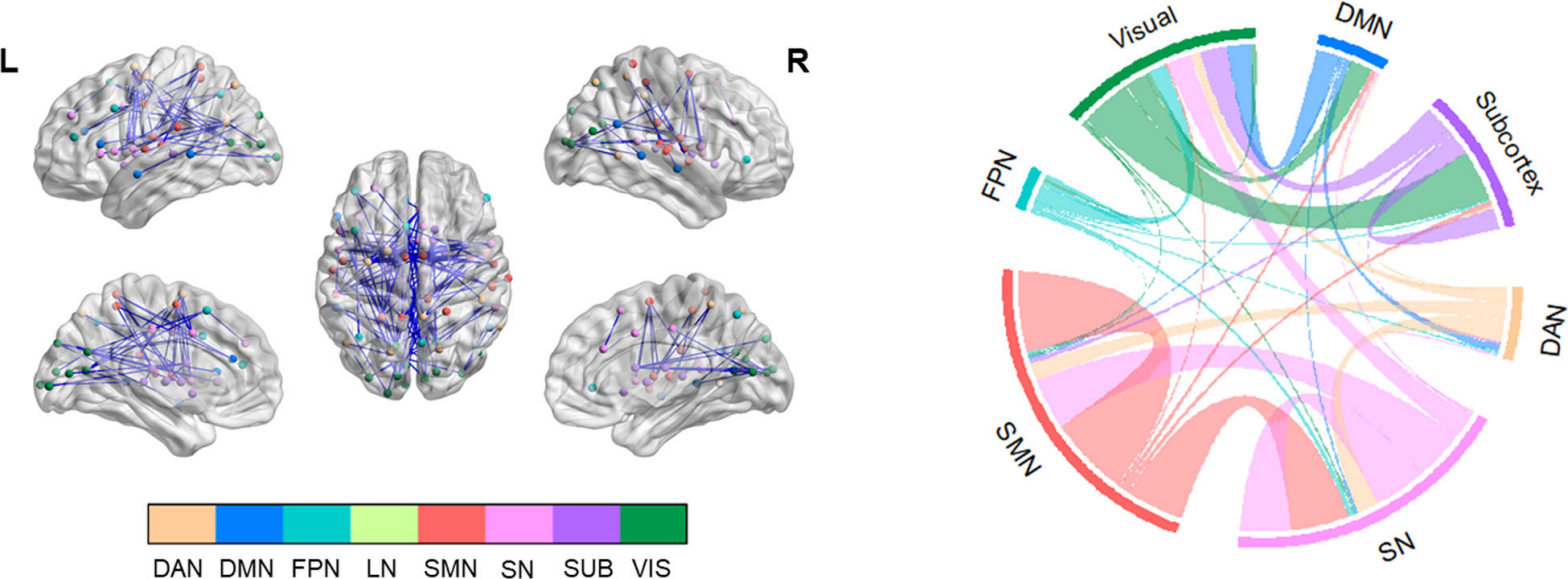
Task-dependent functional connectivity changes during emotion regulation (regulate negative > look negative). Left panel: Results were visualized on brain surface. Each network node is assigned to a specific color. Blue edge color represents negative associations. Right panel: Each network is assigned to a specific color. Bundle color in the chord diagram represents directionality. Connections arising from the source region are depicted with the color of the source region. All results were corrected with network-based statistics (*p* < 0.001).

During emotion regulation, we found decreases in functional connectivity in several brain regions (*p* < 0.001, NBS-corrected). In total, 225 connections were related to task effect. Most of these task-related alterations were between visual, frontal, parietal, and sensory-motor areas, corresponding to visual network, salience network (SN), and sensory-motor network (Fig. 1). Visual network received input/signal from higher cognitive networks such as default-mode network (DMN), SN, and frontoparietal network (FPN) and sent input to subcortex. These negative influences from high-order networks might be related to reduced visual and attentional processing of negative images during emotion regulation. The full list of connections with t values and effect sizes can be found in Table S3.

### Developmental stress and task-dependent functional connectivity

#### Prenatal stress

**Fig. 2:**
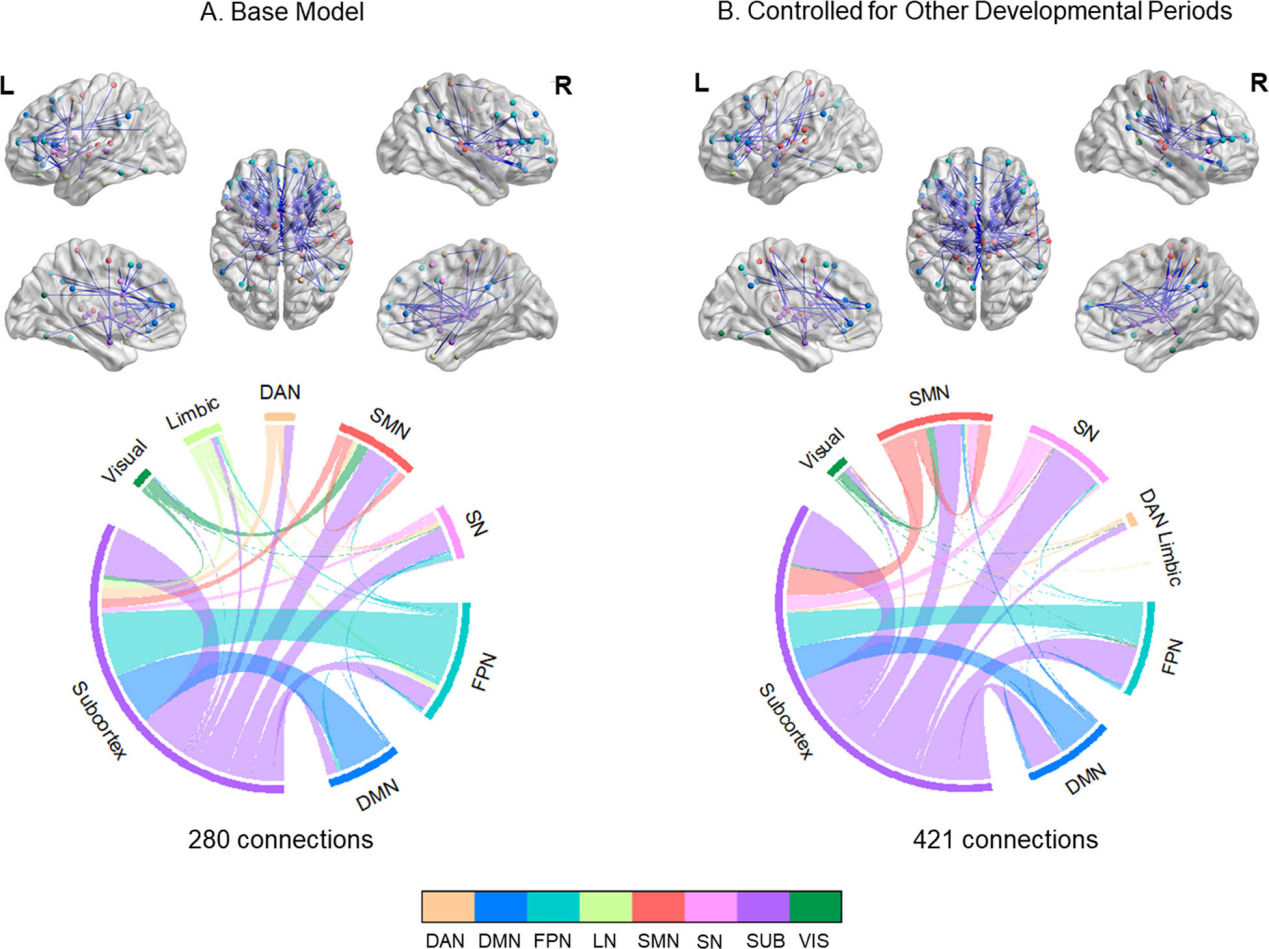
Negative associations between prenatal stress and functional connectivity changes during emotion regulation. Panel **A**. illustrates the connectivity changes that were controlled for the covariates of interest (sex, current life stress, current psychopathology and mean FD), while panel **B**. illustrates the connectivity changes that were additionally controlled for stress occurring at other developmental periods. Upper panel: Results were visualized on brain surface. Each network node is assigned to a specific color. Blue edge color represented negative associations. Lower panel: Each network is assigned to a specific color. Bundle color in the chord diagrams represents directionality. Connections arising from the source region are depicted with the color of the source region. All results were corrected with network-based statistics (*p* < 0.001).

Prenatal and newborn stress was negatively associated with functional connectivity (280 connections; *p* < 0.001, NBS-corrected), mostly in subcortical and frontal regions. Specifically, outgoing connections from frontal regions to subcortex and cingulum and outgoing connections from subcortex to other networks were affected. Subcortical connections included thalamus (incoming and outgoing), striatum (mostly incoming), and to-smaller-extent amygdala. At the network level, these alterations corresponded to outgoing connections from FPN, dorsal attention network (DAN), and DMN to subcortex and outgoing connections from subcortex to several networks (Fig. 2A, Table S4). These alterations did not overlap with main task effects and showed small to medium effect sizes (Hedge’s g = [−0.40, −0.28]). Results were similar when the stress occurring at other developmental periods was controlled (Fig. 2B).

#### Infancy and toddlerhood stress

We did not identify any association between life stress during infancy/toddlerhood and functional connectivity during emotion regulation at corrected level.

#### Childhood stress

**Fig. 3:**
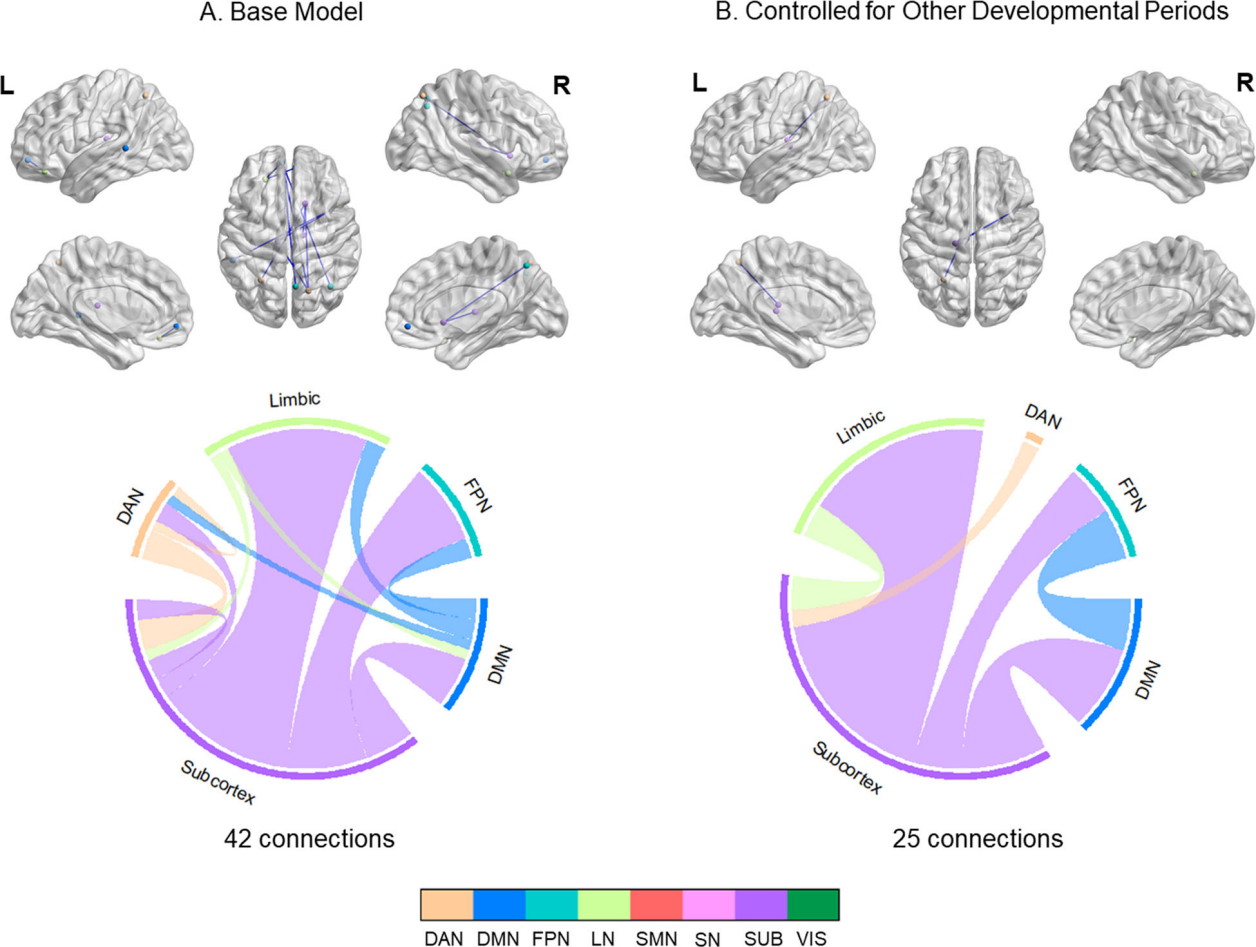
Negative associations between childhood stress and functional connectivity changes during emotion regulation. Panel **A**. illustrates the connectivity changes that were controlled for the covariates of interest (sex, current life stress, current psychopathology and mean FD), while panel **B**. illustrates the connectivity changes that were additionally controlled for stress occurring at other developmental periods. Upper panel: Results were visualized on brain surface. Each network node is assigned to a specific color. Blue edge color represented negative associations. Lower panel: Each network is assigned to a specific color. Bundle color in the chord diagrams represents directionality. Connections arising from the source region are depicted with the color of the source region. All results were corrected with network-based statistics (*p* < 0.001).

Childhood stress was negatively associated with functional connectivity (42 connections; *p* < 0.001, NBS-corrected) in subcortical, temporal and parietal regions (Table S6). Subcortical regions included mostly thalamus. These alterations corresponded to connections from subcortex to several networks including limbic, DMN and attention networks (FPN, DAN) (Fig. 3A). These alterations did not overlap with main task effects and showed small effect sizes (Hedge’s g = [−0.22, 0.17]). Similar results were identified when the stress occurring at other developmental periods was controlled (Fig. 3B). However, those changes were to a lesser extent (*n* = 25), mostly covering the connections from subcortex to limbic network and DMN.

#### Adolescence stress

**Fig. 4:**
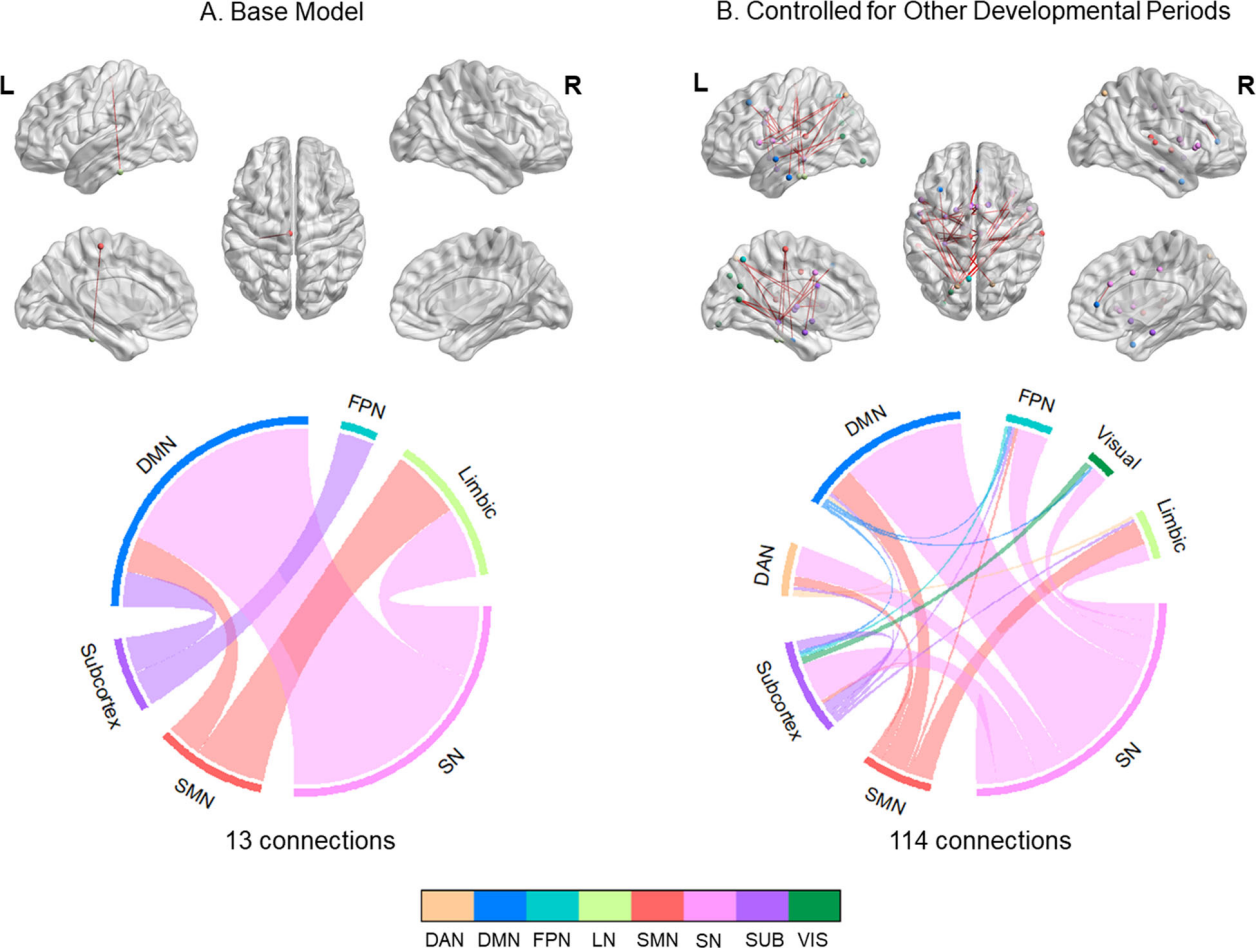
Positive associations between adolescence stress and functional connectivity changes during emotion regulation. Panel **A**. illustrates the connectivity changes that were controlled for the covariates of interests (sex, current life stress, current psychopathology and mean FD), while panel **B**. illustrates the connectivity changes that were additionally controlled for stress occurring at other developmental periods. Upper panel: Results were visualized on brain surface. Each network node is assigned to a specific color. Red edge color represented positive associations. Lower panel: Each network is assigned to a specific color. Bundle color in the chord diagrams represents directionality. Connections arising from the source region are depicted with the color of the source region. All results were corrected with network-based statistics (*p* < 0.001).

Few connections (*n* = 13; *p* < 0.001, NBS-corrected) were positively related to adolescence stress (Fig. 4A, Table S8). These alterations did not overlap with main task effects, showed small effect sizes (Hedge’s g = [0.18, 0.22]), and were not replicated in other atlases. However, when controlling for earlier stress exposure, we identified a large number of changes (*n* = 114) encompassing the connections from cingulum to other regions (Fig. S14), which corresponded to the connections from SN to cognitive networks (DMN, FPN, DAN) (Fig. 4B).

### Brain-behavior relationship

Although we identified some associations between connectivity changes and psychopathology measures, these associations were small and did not survive the FDR correction (Table S9).

### Sensitivity analyses

#### Brain parcellation

For prenatal and childhood stress, we found similar connectivity changes across the atlases (Fig. S9–S12). We found only a few alterations related to adolescence stress (Fig. S13), which were not replicated in other atlases. However, when we controlled the impact of other developmental periods, we obtained similar results for adolescence stress across the atlases (Fig. S14).

#### Impact of self-report

Results remained similar when we used adolescence stress scores calculated solely based on self-reports (Fig. S15).

#### Regulation strategy

Excluded participants had higher scores in adolescence stress compared to included participants (z = 3.28, *p* = 0.001). Results were replicated for prenatal stress and adolescence stress (Fig. S16–S17), although the latter was replicated only when the impact of other developmental periods was controlled and involved less number of connectivity alterations from SN. We did not identify any association between childhood stress and functional connectivity in reduced sample despite the lack of association between childhood stress and reappraisal use during the task.

### Habitual use of emotion regulation strategies

Habitual use of suppression was associated with higher internalizing symptoms (rs = 0.36, FDR-*p* < 0.001), lower emotion regulation success (r = −0.23, FDR-*p* = 0.03), and higher adolescence stress (r = 0.22, FDR-*p* = 0.02). Both higher adolescent stress (r = −0.18, FDR-*p* = 0.04) and higher suppression (r = −0.24, FDR-*p* = 0.02) were related to lower ratings of negative affect during viewing of negative images. No association was found for habitual use of reappraisal.

## Discussion

Here, we investigated long-term associations between ELS and whole-brain functional connectivity by adopting a developmental perspective. Our results suggested that the impact of stress on adult brain connectivity embodied developmentally specific patterns. During emotion regulation, higher prenatal and childhood stress were related to lower functional connectivity between subcortical regions and cognitive networks, while higher adolescence stress was associated with higher connectivity from SN to subcortex and cognitive networks. Our findings suggest that ELS can be linked to connectivity alterations in the adult brain, covering cognitive, limbic, and subcortical networks that are important for emotion regulation [16].

Our results were compatible with a previous study identifying lower functional connectivity in adults exposed to prenatal maternal anxiety [33], supporting that prenatal stress might indeed be related to long-term changes in brain connectivity. The brain is highly plastic early in life [64] and capable of changing its organization to meet environmental requirements [65]. Functional network organization already starts in utero and continues to mature across development [66–69]. Given the existence of network formation, environmental stressors might potentially alter the functional organization of the brain very early in life. In line with this, fetal programming hypothesis posits that environmental stressors during sensitive windows of fetal development can exert long-lasting influences on health [70]. However, exposure to prenatal stress might be result in adaptive responses, especially when prenatal and postnatal environments are matched in terms of stress, since it gives the opportunity to prepare the developing organism for future challenges [64].

Most of the alterations identified for prenatal stress exposure encompassed the connections involving thalamus and striatum, suggesting that connectivity of subcortical regions might be vulnerable to ELS. In line with this, human neuroimaging studies conducted in fetuses and infants provided evidence for altered connectivity of subcortical regions (e.g., amygdala, hippocampus, thalamus) with regard to prenatal stress [64]. We here measured stimulus-based stress exposure rather than perceived/transactional-based stress. Specifically, the studies focusing on more objective aspect of prenatal stress identified altered thalamic connectivity in infants [29, 30]. In line with this, we identified widespread alterations in the thalamo-cortico-striatal pathway, which is essential for learning, behavioral flexibility, attention shifting across cognitive, limbic and sensorimotor modalities [71, 72].

Similar to prenatal stress, childhood stress involved the altered connectivity of subcortex. However, while prenatal stress was related to altered incoming connections to subcortical regions, especially from frontal cortex, childhood stress was mostly related to altered outgoing connections from subcortex to temporal regions. Likewise, most of the subcortical connections stem from the thalamus. The thalamus exhibits a protracted trajectory and reaches maturation slower than other subcortical structures [73]. There are also substantial differences in its connectivity across development [74], which can explain the extended vulnerability of this region to environmental stressors. Additionally, we found that childhood stress was associated with increased activation in the left fusiform gyrus — an occipito-temporal region, which might reflect enhanced visual processing during emotion regulation.

We found no consistent neural marker of adolescence stress when we used total exposure scores embodying shared variance with earlier periods. However, we identified a large number of connectivity changes when we controlled the impact of earlier developmental periods. Moreover, distinct from prenatal and childhood stress, stress unique to adolescence exhibited positive associations with task-specific connectivity. The network alterations mostly encompassed increased connectivity from SN to subcortex and cognitive networks. SN is involved in detecting of salient stimuli [75] and plays an important role in mediating interaction between emotion perception and executive control [76]. Therefore, enhanced information flow from SN to subcortex and cognitive networks might potentially reflect heightened awareness of emotional stimuli and increased demand to regulate. This is also in line with our behavioral results suggesting that adolescence stress is associated with lower emotion regulation success (i.e., smaller decrease in negative affect). Adolescence is characterized by enhanced sensitivity to emotional and social stimuli [77] and ongoing maturation of cognitive control regions [78]. Therefore, stress occurring specifically in this period might alter the interactions between the networks involving detection and regulation of emotional stimuli. Taken together, these results might indicate that functional connectivity might be more sensitive to stress occurring in earlier periods [28], however, when disentangled from earlier stress, adolescence stress might show unique patterns of associations with functional connectivity.

Importantly, both childhood and adolescent stress were related to higher psychopathology symptoms during adulthood. Adolescence stress was further associated with lower regulation success during the task and higher habitual use of suppression. Our supplemental analysis revealed that lower regulation success can be due to lower ratings of negative affect during passive viewing of negative images, which might be explained by higher use of suppression. In addition, stress-related connectivity changes showed small associations with psychopathology measures at uncorrected level. Although a small portion of stress-related neural changes indicated vulnerability direction, associations were not large enough to draw conclusions about their behavioral relevance.

The current study has several limitations. First, we measured life stress using predominantly parental reports and used a cumulative stress approach without addressing some important dimensions of stress including chronicity, specificity, and subjective perspective. Future studies are necessary to identify neural alterations related to other important aspects of stress exposure. Second, the time span for the developmental stages was based on available data in this study. They might not reflect the exact stage of development. Third, recall bias in reporting stressful events may arise due to the length of the measurement intervals (average 34 months, range: 12 months to 4 years). However, life events were measured via a standard interview procedure, which included follow-up questions and verification of timing information using responses from previous waves. These strategies helped to reduce the potential for recall bias. Fourth, our data is suitable for investigating long-term associations only. Longitudinal neuroimaging approaches are necessary to address the temporal dynamics of relationships to infer causality. Fifth, although we attempted to reduce bias in our findings by using multiple parcellation schemes, there are still several methodological issues that need to be addressed by future studies, such as developing advanced correction techniques for multiple testing correction.

This study highlighted the distinct effects of developmental stress exposure on functional connectivity. Prenatal and childhood stress exhibited overlapping alterations in functional connectivity, characterized by lower connectivity between subcortical regions and cognitive networks, while also demonstrating unique, time-specific alterations. In contrast, adolescence stress was related to functional connectivity changes primarily when its effects were isolated from earlier stress exposures, suggesting the importance of considering cumulative and timing-specific stress effects. These findings contribute to our understanding of how stress during specific developmental periods impacts brain function, which may have implications for identifying critical windows for intervention to mitigate long-term mental health risks.

## Supplementary information

Supplementary Material

## Data Availability

The code used in this study is available upon reasonable request from the corresponding author.
