# Supplementary material for: The long-term correlates of developmental stress on whole-brain functional connectivity during emotion regulation
Source: Transl Psychiatry. 2025 Apr 18;15:152. doi: 10.1038/s41398-025-03374-8 (PMC12008206; doi:10.1038/s41398-025-03374-8)
Supplement: Supplementary file 1 — Supplementary Material [file 41398_2025_3374_MOESM1_ESM.docx]

Supplementary Material to

*The long-term correlates of developmental stress on whole-brain functional connectivity during emotion regulation*

Seda Sacu^1,2^, Andrea Hermann^3,4,5^, Tobias Banaschweski^1,2^, Martin F. Gerchen^2,6,7 †^, Nathalie E. Holz^1,2 †^

^1^ Department of Child and Adolescent Psychiatry and Psychotherapy, Central Institute of Mental Health, Medical Faculty Mannheim, University of Heidelberg, Mannheim, Germany

^2^ German Center for Mental Health (DZPG), partner site Mannheim-Heidelberg-Ulm, Germany

^3^ Department of Psychotherapy and Systems Neuroscience, Justus Liebig University, Giessen, Germany

^4^ Bender Institute of Neuroimaging, Justus Liebig University, Giessen, Germany

^5^ Center for Mind, Brain and Behavior, Phillips University Marburg and Justus Liebig University, Giessen, Germany

^6^ Department of Clinical Psychology, Central Institute of Mental Health, Medical Faculty Mannheim, University of Heidelberg, Mannheim, Germany

^7^ Department of Psychology, University of Heidelberg, Heidelberg, Germany

^†^ equal contribution

Table of Contents

[Methods and Materials 3](#_Toc184058309)

[S1. Study Design 3](#_Toc184058310)

[S2. Stressful Life Events 4](#_Toc184058311)

[S3. Experimental Paradigm 6](#_Toc184058312)

[S4. Brain-Behavior Relationship 7](#_Toc184058313)

[Results 8](#_Toc184058314)

[S5.Task-Related Brain Activation 8](#_Toc184058315)

[S6. Developmental Stress and Task-Related Brain Activation 9](#_Toc184058316)

[S7. Task-Dependent Functional Connectivity 10](#_Toc184058317)

[S8. Developmental Stress and Task-Dependent Functional Connectivity 15](#_Toc184058318)

[S9. Brain-Behavior Relationship 30](#_Toc184058319)

[S10. Sensitivity Analysis 31](#_Toc184058320)

[S10.1. Brain Parcellation 31](#_Toc184058321)

[S10.2. Impact of Self-Report 38](#_Toc184058322)

[S10.3. Regulation Strategy 40](#_Toc184058323)

# Methods and Materials

## S1. Study Design

Mannheim Study of Children at Risk is a longitudinal birth cohort study designed to investigate long-term outcomes of early psychosocial and biological risk factors on development (Laucht et al., 2000). The initial sample included 384 children born between 1986 and 1988. The participants were followed from their birth up to the age of 33 years across 11 assessment waves. Across the assessment waves, several measures of adversity, psychopathology, and socio-emotional behavior were collected alongside biological, neurophysiological, and neuroimaging data. Figure S1 illustrates only the assessments used for the current study.

At the last assessment wave, 256 (67%) participants agreed to participate in the study. Among them, 170 participants completed several task-based fMRI paradigms in social and emotional domains.


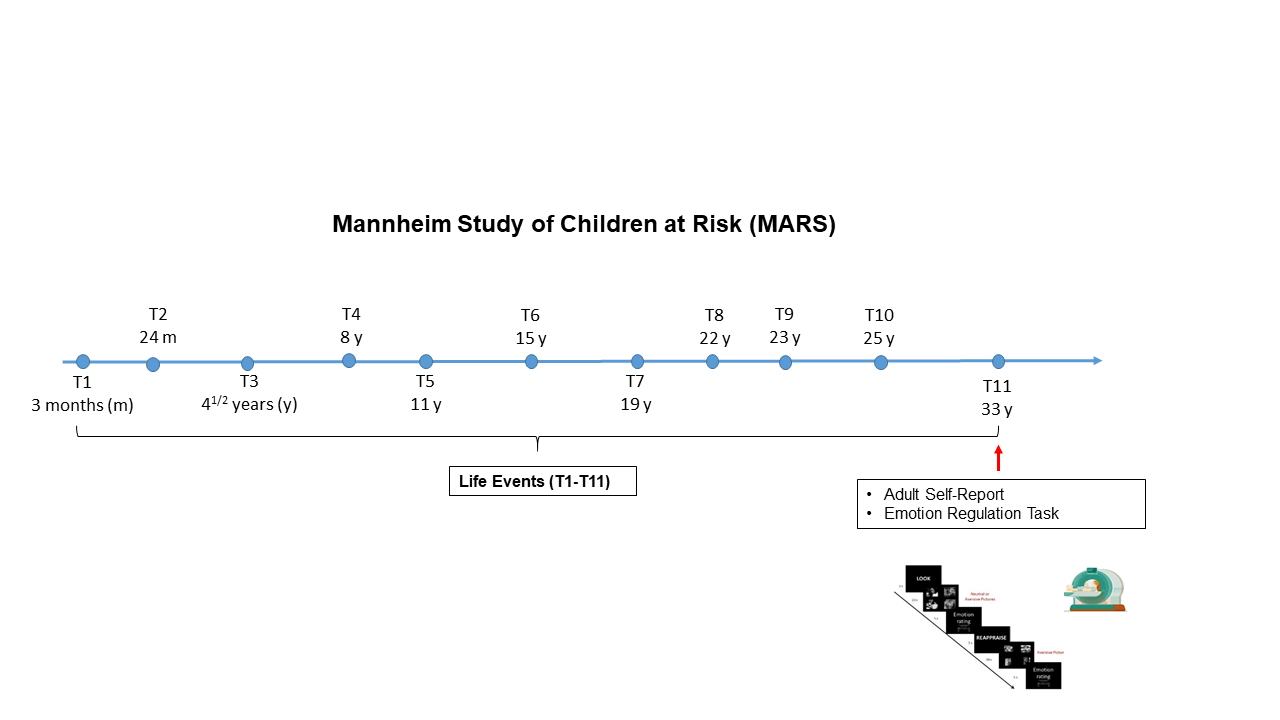


**Figure S1.** Design of Mannheim Study of Children at Risk.

## S2. Stressful Life Events

Stressful life events were measured using a modified version of the Munich Event List (Maier-Diewald et al., 1983). The items covered positive and negative stressors in several domains, including partnership, education, work, health, and finance. At the first assessment wave, parents were asked to report occurrence of life events in the last year, which covered prenatal and newborn period. From T2 onward, life events were recorded if they occurred between the previous assessment wave and the current assessment wave. Between T1 and T6 (15 years), trained psychologists conducted a standardized interview with caregivers. The caregiver reported occurrence and frequency of each event. Starting at the 15-year assessment, participants rated stressful life events themselves. However, since the adolescent version at T6 was a short version and did not cover all domains, we opted to use the parent version for compatibility. At T7, only occurrence is counted, no information regarding frequency was collected. T11 measure covered the events that occurred in the last 12 months.

**Table S1.** Stressful Life Events.

|  | T1 | T2 | T3 | T4 | T5 | T6 | T7 | T11 |
| --- | --- | --- | --- | --- | --- | --- | --- | --- |
| Item number | 41 | 42 | 44 | 47 | 47 | 50 | 53 | 57 |
| Mean (SD) | 3.80(2.49) | 5.58(3.20) | 6.63(3.35) | 6.72(3.54) | 5.57(3.40) | 6.37(3.73) | 7.22(4.55) | 4.07(3.45) |
| Score range | 1-15 | 0-21 | 2-17 | 0-23 | 0-16 | 0-22 | 0-28 | 0-18 |


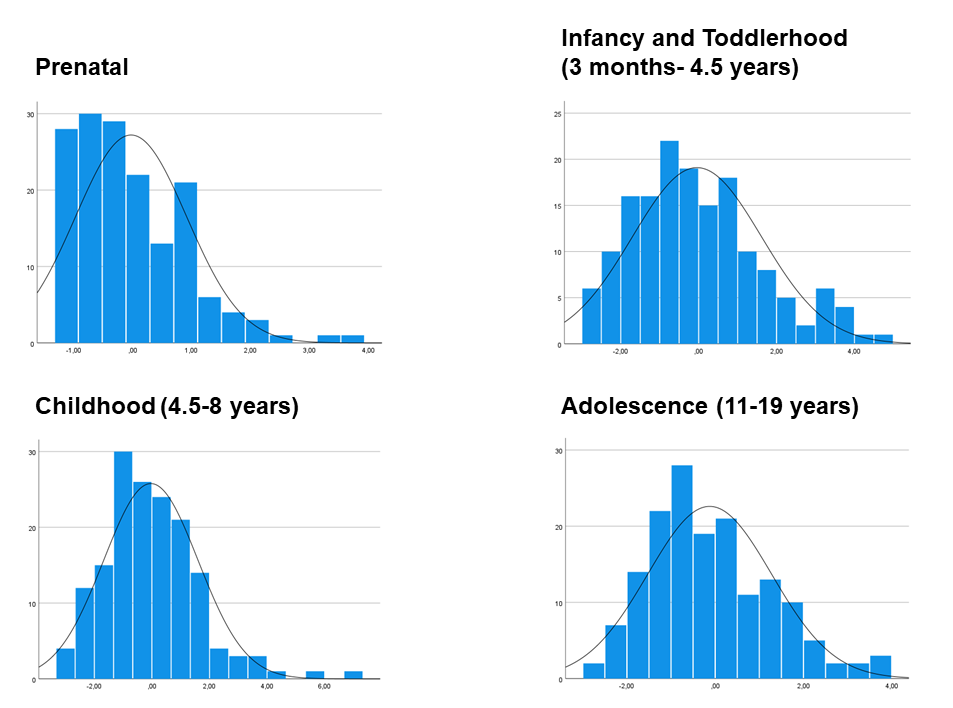


**Figure S2.** Data Distribution.

**Table S2.** Spearman’s correlation between life stress variables.

|  | Prenatal | Infancy/ Toddlerhood | Childhood | Adolescence | Current (T11) |
| --- | --- | --- | --- | --- | --- |
| Prenatal | - | 0.42** | 0.30** | 0.26* | 0.07 |
| Infancy/ Toddlerhood |  | - | 0.43** | 0.30** | 0.15 |
| Childhood |  |  | - | 0.45** | 0.06 |
| Adolescence |  |  |  | - | 0.08 |
| Current (T11) |  |  |  |  | - |

*p<0.01 **p< 0.001

## S3. Experimental Paradigm

Each block started with a 3 s instruction (e.g., Look or Reappraise). Participants subsequently viewed a 20 s block of neutral or negative images from the International Affective Picture System (Lang et al., 2008). Each image was presented for 5 s consecutively without an interstimulus interval. Immediately following the experimental block, participants were asked to rate the intensity of their negative affect on a 7-point Likert scale (1 = no negative feelings at all; 7 = extremely negative feelings) via a button press (max 4 s). A white fixation cross on black background was presented during the inter-trial interval up to a total block duration of 30 s. The total task comprised four blocks per condition (12 blocks in total) and lasted for 6 min 37 s. The blocks were randomly presented in four runs with a maximum of two presentations of the same condition in succession.


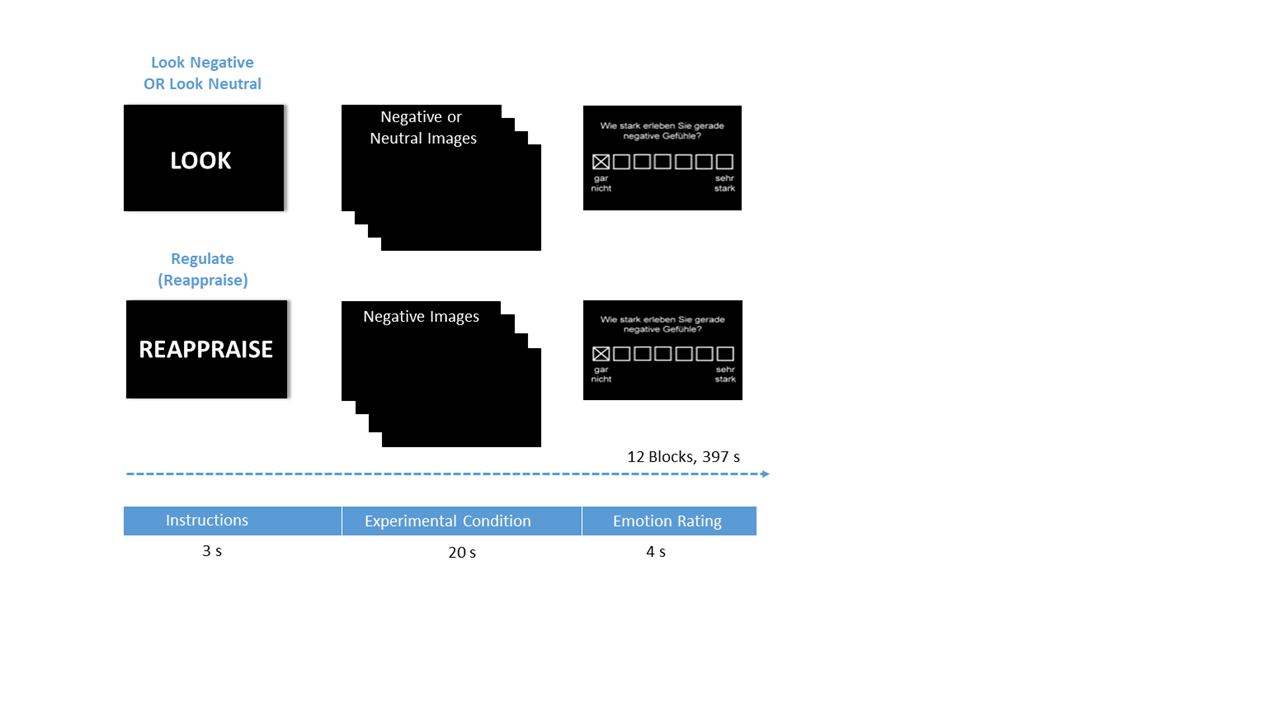


**Figure S3.** Emotion Regulation Task.

## S4. Brain-Behavior Relationship

Simple linear regression using the ordinary least square approach was conducted to see if altered connectivity was linked to any psychopathology measure (internalizing or externalizing symptoms). For the model containing baseline set of covariates (model 1), the test is conducted for 280, 42, and 10 connections related to prenatal, childhood and adolescence stress respectively. For the model controlling the impact of other developmental periods (model 2), the test is conducted for 421, 25, and 114 connections related to prenatal, childhood and adolescence stress respectively. Connections parameter showing a significant association (uncorrected-p < 0.05) was reported in the Table S9 with their coefficients and adjusted R^2^ scores.

# Results

## S5.Task-Related Brain Activation


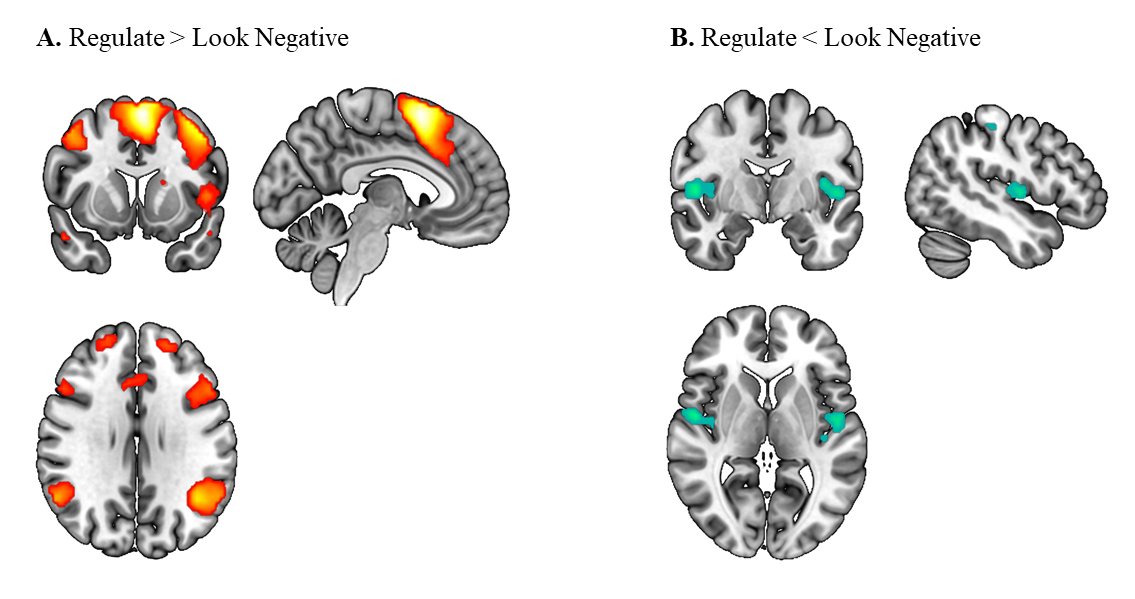


**Figure S4.** Task-related activation during emotion regulation task. **(A)** Brain regions showing increased activation during the emotion regulation condition compared to the look negative condition were mapped on brain surface in hot colors using MRIcroGL (<https://www.nitrc.org/projects/mricrogl>). **(B)** Brain regions showing decreased activation during the emotion regulation condition compared to the look negative condition were mapped on brain surface in cold colors. P < 0.05 (FWE-corrected).

## S6. Developmental Stress and Task-Related Brain Activation


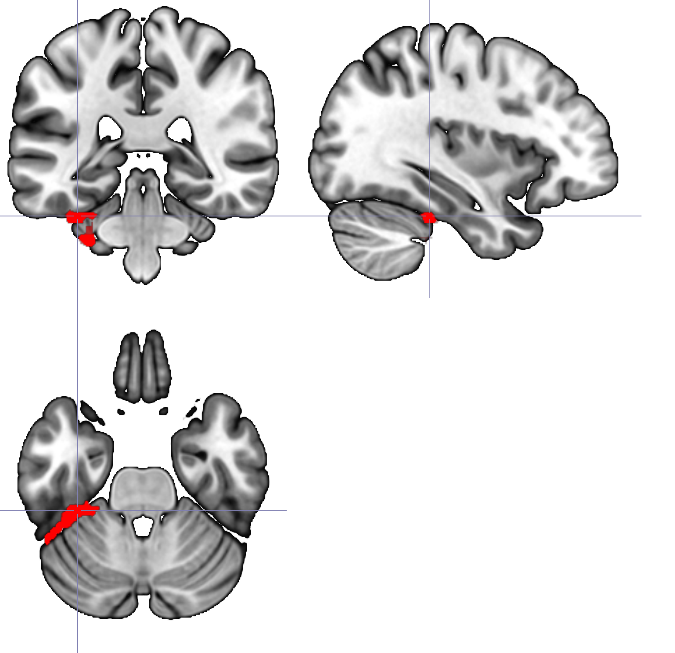


**Figure S5.** The association between childhood stress and brain activation during emotion regulation. Cluster depicted with the red color included left fusiform and cerebellum (k=83, t=4.52, p < 0.05 cluster-level FWE corrected).

## S7. Task-Dependent Functional Connectivity

**Table S3.** Task-dependent functional connectivity during emotion regulation (Network-Based Statistic (NBS)-corrected).

| **Connection** | **Seed Region** | **Target Region** | **Seed Network** | **Target Network** | **t** | **Hedge’s g** |
| --- | --- | --- | --- | --- | --- | --- |
| 1 | SFG_L_7_4 | SFG_L_7_5 | DAN | SMN | -3.65 | -0.29 |
| 2 | PrG_L_6_5 | SFG_L_7_5 | SN | SMN | -3.92 | -0.31 |
| 3 | SPL_L_5_4 | SFG_L_7_5 | SMN | SMN | -3.96 | -0.31 |
| 4 | PCun_L_4_2 | SFG_L_7_5 | SMN | SMN | -4.11 | -0.32 |
| 5 | INS_L_6_6 | SFG_L_7_5 | SN | SMN | -3.85 | -0.30 |
| 6 | CG_L_7_5 | SFG_L_7_5 | SN | SMN | -3.99 | -0.31 |
| 7 | CG_R_7_5 | SFG_L_7_5 | SN | SMN | -4.22 | -0.33 |
| 8 | SPL_L_5_4 | SFG_R_7_5 | SMN | SMN | -3.95 | -0.31 |
| 9 | PCun_L_4_2 | SFG_R_7_5 | SMN | SMN | -3.57 | -0.28 |
| 10 | INS_L_6_6 | SFG_R_7_5 | SN | SMN | -3.56 | -0.28 |
| 11 | CG_L_7_5 | SFG_R_7_5 | SN | SMN | -3.67 | -0.29 |
| 12 | CG_R_7_5 | SFG_R_7_5 | SN | SMN | -3.96 | -0.31 |
| 13 | SFG_L_7_1 | MFG_L_7_1 | FPN | SN | -3.83 | -0.30 |
| 14 | PCun_R_4_3 | IFG_L_6_1 | VIS | FPN | -3.49 | -0.27 |
| 15 | MVOcC _L_5_3 | OrG_R_6_1 | VIS | DMN | -4.12 | -0.32 |
| 16 | LOcC_L_4_3 | OrG_R_6_1 | VIS | DMN | -3.86 | -0.30 |
| 17 | LOcC _R_2_1 | OrG_R_6_1 | VIS | DMN | -3.60 | -0.28 |
| 18 | SFG_L_7_4 | PrG_L_6_5 | DAN | SN | -3.85 | -0.30 |
| 19 | SFG_L_7_5 | PrG_L_6_5 | SMN | SN | -4.52 | -0.35 |
| 20 | SFG_R_7_5 | PrG_L_6_5 | SMN | SN | -4.01 | -0.31 |
| 21 | PCL_R_2_1 | PrG_L_6_5 | SMN | SN | -3.80 | -0.30 |
| 22 | IPL_L_6_6 | PrG_L_6_5 | SMN | SN | -3.59 | -0.28 |
| 23 | INS_L_6_6 | PrG_L_6_5 | SN | SN | -3.66 | -0.29 |
| 24 | INS_R_6_6 | PrG_L_6_5 | SN | SN | -4.09 | -0.32 |
| 25 | CG_L_7_5 | PrG_L_6_5 | SN | SN | -4.19 | -0.33 |
| 26 | CG_R_7_5 | PrG_L_6_5 | SN | SN | -3.98 | -0.31 |
| 27 | CG_R_7_6 | PrG_L_6_5 | SN | SN | -3.70 | -0.29 |
| 28 | SFG_L_7_4 | PrG_R_6_5 | DAN | SN | -3.70 | -0.29 |
| 29 | SFG_L_7_5 | PrG_R_6_5 | SMN | SN | -4.78 | -0.37 |
| 30 | SFG_R_7_5 | PrG_R_6_5 | SMN | SN | -4.57 | -0.36 |
| 31 | IFG_L_6_6 | PrG_R_6_5 | SN | SN | -3.54 | -0.28 |
| 32 | PrG_L_6_2 | PrG_R_6_5 | DAN | SN | -3.76 | -0.30 |
| 33 | PrG_L_6_5 | PrG_R_6_5 | SN | SN | -3.62 | -0.28 |
| 34 | PCL_R_2_1 | PrG_R_6_5 | SMN | SN | -3.66 | -0.29 |
| 35 | STG_R_6_3 | PrG_R_6_5 | SMN | SN | -3.66 | -0.29 |
| 36 | IPL_L_6_6 | PrG_R_6_5 | SMN | SN | -4.20 | -0.33 |
| 37 | PCun_L_4_2 | PrG_R_6_5 | SMN | SN | -3.52 | -0.28 |
| 38 | INS_L_6_6 | PrG_R_6_5 | SN | SN | -4.20 | -0.33 |
| 39 | INS_R_6_6 | PrG_R_6_5 | SN | SN | -4.68 | -0.37 |
| 40 | CG_L_7_5 | PrG_R_6_5 | SN | SN | -4.67 | -0.37 |
| 41 | CG_R_7_5 | PrG_R_6_5 | SN | SN | -4.39 | -0.34 |
| 42 | CG_L_7_6 | PrG_R_6_5 | SN | SN | -3.76 | -0.29 |
| 43 | CG_R_7_7 | PCL_R_2_1 | DMN | SMN | -3.62 | -0.28 |
| 44 | PCun_L_4_2 | STG_L_6_2 | SMN | SMN | -3.70 | -0.29 |
| 45 | CG_R_7_6 | STG_L_6_2 | SN | SMN | -4.39 | -0.34 |
| 46 | PCL_R_2_1 | STG_R_6_2 | SMN | SMN | -3.64 | -0.29 |
| 47 | PCun_L_4_2 | STG_R_6_2 | SMN | SMN | -3.77 | -0.30 |
| 48 | CG_L_7_6 | STG_R_6_2 | SN | SMN | -3.56 | -0.28 |
| 49 | CG_R_7_6 | STG_R_6_2 | SN | SMN | -3.87 | -0.30 |
| 50 | PCun_L_4_2 | STG_L_6_3 | SMN | SMN | -3.66 | -0.29 |
| 51 | SFG_L_7_5 | STG_R_6_3 | SMN | SMN | -3.72 | -0.29 |
| 52 | PCL_R_2_1 | STG_R_6_3 | SMN | SMN | -3.67 | -0.29 |
| 53 | SPL_L_5_4 | STG_R_6_3 | SMN | SMN | -3.52 | -0.28 |
| 54 | PCun_L_4_2 | STG_R_6_3 | SMN | SMN | -4.29 | -0.34 |
| 55 | STG_R_6_2 | STG_R_6_4 | SMN | SMN | -3.84 | -0.30 |
| 56 | LOcC_L_4_3 | MTG_L_4_4 | VIS | DMN | -3.71 | -0.29 |
| 57 | IFG_L_6_6 | SPL_L_5_2 | SN | DAN | -3.57 | -0.28 |
| 58 | MFG_L_7_2 | IPL_L_6_2 | FPN | FPN | -3.54 | -0.28 |
| 59 | MFG_L_7_4 | IPL_R_6_3 | FPN | DAN | -3.52 | -0.28 |
| 60 | SFG_L_7_4 | IPL_L_6_6 | DAN | SMN | -3.55 | -0.28 |
| 61 | SFG_L_7_5 | IPL_L_6_6 | SMN | SMN | -4.02 | -0.32 |
| 62 | SFG_R_7_5 | IPL_L_6_6 | SMN | SMN | -4.10 | -0.32 |
| 63 | PrG_L_6_2 | IPL_L_6_6 | DAN | SMN | -3.81 | -0.30 |
| 64 | PrG_L_6_5 | IPL_L_6_6 | SN | SMN | -3.69 | -0.29 |
| 65 | PCL_R_2_1 | IPL_L_6_6 | SMN | SMN | -3.64 | -0.29 |
| 66 | PCun_L_4_2 | IPL_L_6_6 | SMN | SMN | -3.57 | -0.28 |
| 67 | INS_L_6_6 | IPL_L_6_6 | SN | SMN | -3.53 | -0.28 |
| 68 | CG_L_7_5 | IPL_L_6_6 | SN | SMN | -3.63 | -0.28 |
| 69 | CG_R_7_5 | IPL_L_6_6 | SN | SMN | -3.87 | -0.30 |
| 70 | CG_L_7_6 | IPL_L_6_6 | SN | SMN | -3.55 | -0.28 |
| 71 | CG_R_7_6 | IPL_L_6_6 | SN | SMN | -4.30 | -0.34 |
| 72 | SFG_L_7_5 | IPL_R_6_6 | SMN | SMN | -3.98 | -0.31 |
| 73 | SFG_R_7_5 | IPL_R_6_6 | SMN | SMN | -4.19 | -0.33 |
| 74 | INS_L_6_6 | IPL_R_6_6 | SN | SMN | -3.88 | -0.30 |
| 75 | CG_R_7_5 | IPL_R_6_6 | SN | SMN | -3.98 | -0.31 |
| 76 | CG_L_7_6 | IPL_R_6_6 | SN | SMN | -3.81 | -0.30 |
| 77 | CG_R_7_6 | IPL_R_6_6 | SN | SMN | -3.64 | -0.29 |
| 78 | MFG_L_7_4 | PCun_R_4_1 | FPN | FPN | -3.86 | -0.30 |
| 79 | STG_R_6_6 | PCun_R_4_2 | DMN | DAN | -3.69 | -0.29 |
| 80 | CG_L_7_3 | PCun_R_4_2 | DMN | DAN | -3.60 | -0.28 |
| 81 | CG_R_7_7 | PCun_R_4_2 | DMN | DAN | -3.77 | -0.30 |
| 82 | Tha_L_8_1 | PCun_R_4_2 | SUB | DAN | -3.55 | -0.28 |
| 83 | SFG_L_7_1 | PCun_L_4_3 | FPN | VIS | -3.62 | -0.28 |
| 84 | MFG_L_7_2 | PCun_L_4_3 | FPN | VIS | -3.78 | -0.30 |
| 85 | IFG_L_6_6 | PCun_L_4_3 | SN | VIS | -3.86 | -0.30 |
| 86 | SFG_L_7_1 | PCun_R_4_3 | FPN | VIS | -3.68 | -0.29 |
| 87 | pSTS_R_2_1 | PCun_R_4_3 | DMN | VIS | -3.63 | -0.28 |
| 88 | BG_L_6_5 | PCun_R_4_3 | SUB | VIS | -3.82 | -0.30 |
| 89 | Tha_L_8_4 | PCun_R_4_3 | SUB | VIS | -3.58 | -0.28 |
| 90 | SFG_L_7_5 | PoG_L_4_2 | SMN | SMN | -3.75 | -0.29 |
| 91 | SFG_L_7_1 | PoG_R_4_2 | FPN | SMN | -3.61 | -0.28 |
| 92 | SFG_L_7_5 | PoG_R_4_2 | SMN | SMN | -4.14 | -0.32 |
| 93 | SFG_R_7_5 | PoG_R_4_2 | SMN | SMN | -4.10 | -0.32 |
| 94 | CG_L_7_5 | PoG_R_4_2 | SN | SMN | -3.53 | -0.28 |
| 95 | SPL_L_5_4 | INS_L_6_1 | SMN | SMN | -4 | -0.31 |
| 96 | PCun_L_4_2 | INS_L_6_1 | SMN | SMN | -3.56 | -0.28 |
| 97 | MVOcC _R_5_3 | INS_L_6_1 | VIS | SMN | -3.61 | -0.28 |
| 98 | SFG_L_7_5 | INS_R_6_1 | SMN | SMN | -3.52 | -0.28 |
| 99 | BG_L_6_4 | INS_R_6_1 | SUB | SMN | -3.69 | -0.29 |
| 100 | SFG_L_7_4 | INS_L_6_5 | DAN | SMN | -4.09 | -0.32 |
| 101 | SFG_L_7_5 | INS_L_6_5 | SMN | SMN | -3.83 | -0.30 |
| 102 | SPL_L_5_4 | INS_L_6_5 | SMN | SMN | -3.83 | -0.30 |
| 103 | SFG_L_7_4 | INS_R_6_5 | DAN | SMN | -3.50 | -0.27 |
| 104 | SFG_L_7_5 | INS_R_6_5 | SMN | SMN | -4.32 | -0.34 |
| 105 | SFG_R_7_5 | INS_R_6_5 | SMN | SMN | -4.10 | -0.32 |
| 106 | PrG_L_6_1 | INS_R_6_5 | SMN | SMN | -3.58 | -0.28 |
| 107 | PrG_L_6_2 | INS_R_6_5 | DAN | SMN | -3.70 | -0.29 |
| 108 | PCL_R_2_1 | INS_R_6_5 | SMN | SMN | -3.89 | -0.31 |
| 109 | IPL_L_6_6 | INS_R_6_5 | SMN | SMN | -3.58 | -0.28 |
| 110 | PCun_L_4_2 | INS_R_6_5 | SMN | SMN | -3.80 | -0.30 |
| 111 | PCun_R_4_2 | INS_R_6_5 | DAN | SMN | -3.78 | -0.30 |
| 112 | BG_L_6_3 | INS_R_6_5 | SUB | SMN | -3.56 | -0.28 |
| 113 | SFG_L_7_4 | INS_L_6_6 | DAN | SN | -3.78 | -0.30 |
| 114 | SFG_L_7_5 | INS_L_6_6 | SMN | SN | -3.86 | -0.30 |
| 115 | SFG_R_7_5 | INS_L_6_6 | SMN | SN | -4.03 | -0.32 |
| 116 | IPL_L_6_6 | INS_L_6_6 | SMN | SN | -4.08 | -0.32 |
| 117 | IPL_R_6_6 | INS_L_6_6 | SMN | SN | -3.61 | -0.28 |
| 118 | INS_R_6_6 | INS_L_6_6 | SN | SN | -3.92 | -0.31 |
| 119 | CG_L_7_5 | INS_L_6_6 | SN | SN | -3.77 | -0.30 |
| 120 | CG_R_7_5 | INS_L_6_6 | SN | SN | -4.14 | -0.32 |
| 121 | CG_L_7_6 | INS_L_6_6 | SN | SN | -3.48 | -0.27 |
| 122 | MVOcC _R_5_3 | INS_L_6_6 | VIS | SN | -3.67 | -0.29 |
| 123 | SFG_L_7_5 | INS_R_6_6 | SMN | SN | -3.78 | -0.30 |
| 124 | SFG_R_7_5 | INS_R_6_6 | SMN | SN | -3.97 | -0.31 |
| 125 | IPL_L_6_6 | INS_R_6_6 | SMN | SN | -3.63 | -0.28 |
| 126 | PCun_L_4_2 | INS_R_6_6 | SMN | SN | -3.77 | -0.30 |
| 127 | SPL_L_5_4 | CG_L_7_3 | SMN | DMN | -3.95 | -0.31 |
| 128 | PCun_L_4_2 | CG_L_7_3 | SMN | DMN | -4.13 | -0.32 |
| 129 | PCun_R_4_2 | CG_L_7_3 | DAN | DMN | -3.55 | -0.28 |
| 130 | CG_L_7_6 | CG_L_7_3 | SN | DMN | -3.58 | -0.28 |
| 131 | MVOcC _R_5_3 | CG_L_7_3 | VIS | DMN | -3.85 | -0.30 |
| 132 | LOcC_L_4_3 | CG_L_7_3 | VIS | DMN | -3.71 | -0.29 |
| 133 | SFG_L_7_1 | CG_R_7_3 | FPN | SN | -4.35 | -0.34 |
| 134 | SFG_R_7_1 | CG_R_7_3 | SN | SN | -3.52 | -0.28 |
| 135 | SFG_L_7_4 | CG_R_7_3 | DAN | SN | -4 | -0.31 |
| 136 | PrG_L_6_2 | CG_R_7_3 | DAN | SN | -3.78 | -0.30 |
| 137 | SPL_L_5_4 | CG_R_7_3 | SMN | SN | -3.53 | -0.28 |
| 138 | PCun_L_4_2 | CG_R_7_3 | SMN | SN | -3.84 | -0.30 |
| 139 | CG_L_7_3 | CG_R_7_3 | DMN | SN | -3.53 | -0.28 |
| 140 | CG_L_7_6 | CG_R_7_3 | SN | SN | -3.94 | -0.31 |
| 141 | SFG_L_7_4 | CG_L_7_5 | DAN | SN | -3.68 | -0.29 |
| 142 | SFG_L_7_5 | CG_L_7_5 | SMN | SN | -3.75 | -0.29 |
| 143 | SFG_R_7_5 | CG_L_7_5 | SMN | SN | -3.84 | -0.30 |
| 144 | PrG_L_6_2 | CG_L_7_5 | DAN | SN | -3.74 | -0.29 |
| 145 | SPL_L_5_4 | CG_L_7_5 | SMN | SN | -3.51 | -0.28 |
| 146 | CG_R_7_5 | CG_L_7_5 | SN | SN | -4.21 | -0.33 |
| 147 | CG_L_7_6 | CG_L_7_5 | SN | SN | -3.73 | -0.29 |
| 148 | SFG_L_7_5 | CG_R_7_5 | SMN | SN | -3.63 | -0.28 |
| 149 | PrG_L_6_2 | CG_R_7_5 | DAN | SN | -3.72 | -0.29 |
| 150 | INS_L_6_6 | CG_R_7_5 | SN | SN | -3.60 | -0.28 |
| 151 | LOcC_L_4_3 | CG_L_7_7 | VIS | DMN | -3.98 | -0.31 |
| 152 | MVOcC _L_5_3 | CG_R_7_7 | VIS | DMN | -3.89 | -0.31 |
| 153 | LOcC_L_4_3 | CG_R_7_7 | VIS | DMN | -3.91 | -0.31 |
| 154 | BG_L_6_5 | MVOcC _L_5_2 | SUB | VIS | -3.70 | -0.29 |
| 155 | Tha_L_8_4 | MVOcC _L_5_2 | SUB | VIS | -3.70 | -0.29 |
| 156 | SFG_L_7_1 | MVOcC _R_5_2 | FPN | VIS | -4.13 | -0.32 |
| 157 | IFG_L_6_5 | MVOcC _R_5_2 | SN | VIS | -3.60 | -0.28 |
| 158 | pSTS_L_2_1 | MVOcC _R_5_2 | DMN | VIS | -4.18 | -0.33 |
| 159 | pSTS_R_2_1 | MVOcC _R_5_2 | DMN | VIS | -3.69 | -0.29 |
| 160 | CG_L_7_6 | MVOcC _R_5_2 | SN | VIS | -4.02 | -0.32 |
| 161 | CG_R_7_6 | MVOcC _R_5_2 | SN | VIS | -3.65 | -0.29 |
| 162 | BG_L_6_5 | MVOcC _R_5_2 | SUB | VIS | -3.85 | -0.30 |
| 163 | BG_L_6_5 | MVOcC _R_5_3 | SUB | VIS | -3.49 | -0.27 |
| 164 | SFG_L_7_1 | MVOcC _L_5_5 | FPN | VIS | -4.26 | -0.33 |
| 165 | SFG_R_7_1 | MVOcC _L_5_5 | SN | VIS | -3.75 | -0.29 |
| 166 | PrG_L_6_2 | MVOcC _L_5_5 | DAN | VIS | -3.59 | -0.28 |
| 167 | pSTS_L_2_1 | MVOcC _L_5_5 | DMN | VIS | -3.72 | -0.29 |
| 168 | SPL_R_5_4 | MVOcC _L_5_5 | SMN | VIS | -3.60 | -0.28 |
| 169 | CG_L_7_6 | MVOcC _L_5_5 | SN | VIS | -4.36 | -0.34 |
| 170 | CG_R_7_6 | MVOcC _L_5_5 | SN | VIS | -4.06 | -0.32 |
| 171 | MVOcC _R_5_3 | MVOcC _L_5_5 | VIS | VIS | -3.57 | -0.28 |
| 172 | Tha_L_8_4 | MVOcC _L_5_5 | SUB | VIS | -4.02 | -0.32 |
| 173 | SFG_L_7_1 | MVOcC _R_5_5 | FPN | VIS | -3.94 | -0.31 |
| 174 | pSTS_L_2_1 | MVOcC _R_5_5 | DMN | VIS | -3.61 | -0.28 |
| 175 | Tha_L_8_4 | MVOcC _R_5_5 | SUB | VIS | -3.85 | -0.30 |
| 176 | pSTS_R_2_1 | LOcC_L_4_1 | DMN | VIS | -3.67 | -0.29 |
| 177 | pSTS_R_2_2 | LOcC_L_4_1 | SN | VIS | -4.28 | -0.34 |
| 178 | IPL_L_6_5 | LOcC_L_4_1 | DAN | VIS | -4.38 | -0.34 |
| 179 | BG_L_6_5 | LOcC_L_4_1 | SUB | VIS | -3.51 | -0.28 |
| 180 | MTG_R_4_3 | LOcC_R_4_1 | DAN | VIS | -3.49 | -0.27 |
| 181 | pSTS_R_2_2 | LOcC_R_4_1 | SN | VIS | -3.85 | -0.30 |
| 182 | IPL_R_6_1 | LOcC_R_4_1 | VIS | VIS | -3.98 | -0.31 |
| 183 | IPL_L_6_5 | LOcC_R_4_1 | DAN | VIS | -4.76 | -0.37 |
| 184 | IPL_R_6_5 | LOcC_R_4_1 | DMN | VIS | -3.51 | -0.28 |
| 185 | pSTS_L_2_1 | LOcC_L_4_3 | DMN | VIS | -3.55 | -0.28 |
| 186 | IFG_L_6_3 | LOcC _L_2_1 | DMN | VIS | -3.66 | -0.29 |
| 187 | IFG_R_6_4 | LOcC _L_2_1 | FPN | VIS | -3.57 | -0.28 |
| 188 | IFG_L_6_5 | LOcC _L_2_1 | SN | VIS | -3.72 | -0.29 |
| 189 | STG_L_6_6 | LOcC _L_2_1 | DMN | VIS | -3.61 | -0.28 |
| 190 | BG_L_6_5 | LOcC _L_2_1 | SUB | VIS | -4.17 | -0.33 |
| 191 | Tha_L_8_4 | LOcC _L_2_1 | SUB | VIS | -4.65 | -0.36 |
| 192 | LOcC_L_4_3 | BG_R_6_1 | VIS | SUB | -4.01 | -0.31 |
| 193 | SPL_L_5_4 | BG_L_6_2 | SMN | SUB | -3.52 | -0.28 |
| 194 | PCun_L_4_3 | BG_L_6_2 | VIS | SUB | -3.67 | -0.29 |
| 195 | SPL_L_5_4 | BG_R_6_2 | SMN | SUB | -3.76 | -0.30 |
| 196 | MVOcC _R_5_5 | BG_L_6_4 | VIS | SUB | -3.81 | -0.30 |
| 197 | SPL_R_5_2 | BG_L_6_5 | DAN | SUB | -4 | -0.31 |
| 198 | PCun_R_4_1 | BG_L_6_5 | FPN | SUB | -3.62 | -0.28 |
| 199 | PCun_R_4_3 | BG_L_6_5 | VIS | SUB | -4.27 | -0.34 |
| 200 | MVOcC _L_5_2 | BG_L_6_5 | VIS | SUB | -3.77 | -0.30 |
| 201 | MVOcC _R_5_2 | BG_L_6_5 | VIS | SUB | -4.12 | -0.32 |
| 202 | MVOcC _L_5_3 | BG_L_6_5 | VIS | SUB | -3.97 | -0.31 |
| 203 | MVOcC _R_5_3 | BG_L_6_5 | VIS | SUB | -3.49 | -0.27 |
| 204 | MVOcC _R_5_5 | BG_L_6_5 | VIS | SUB | -3.81 | -0.30 |
| 205 | Tha_R_8_4 | BG_L_6_5 | SUB | SUB | -3.72 | -0.29 |
| 206 | Tha_L_8_6 | BG_L_6_5 | SUB | SUB | -3.51 | -0.28 |
| 207 | PCun_L_4_3 | BG_R_6_5 | VIS | SUB | -3.69 | -0.29 |
| 208 | PCun_R_4_3 | BG_R_6_5 | VIS | SUB | -4.08 | -0.32 |
| 209 | MVOcC _L_5_2 | BG_R_6_5 | VIS | SUB | -4.55 | -0.36 |
| 210 | MVOcC _R_5_2 | BG_R_6_5 | VIS | SUB | -4.80 | -0.38 |
| 211 | MVOcC _L_5_3 | BG_R_6_5 | VIS | SUB | -3.92 | -0.31 |
| 212 | MVOcC _R_5_3 | BG_R_6_5 | VIS | SUB | -3.63 | -0.29 |
| 213 | MVOcC _L_5_5 | BG_R_6_5 | VIS | SUB | -4.01 | -0.31 |
| 214 | MVOcC _R_5_5 | BG_R_6_5 | VIS | SUB | -4.53 | -0.35 |
| 215 | LOcC_L_4_3 | BG_R_6_5 | VIS | SUB | -3.49 | -0.27 |
| 216 | LOcC _L_2_1 | BG_R_6_5 | VIS | SUB | -3.55 | -0.28 |
| 217 | LOcC _R_2_1 | BG_R_6_5 | VIS | SUB | -3.50 | -0.27 |
| 218 | Tha_L_8_6 | BG_R_6_5 | SUB | SUB | -3.60 | -0.28 |
| 219 | LOcC _R_2_1 | Tha_L_8_1 | VIS | SUB | -3.56 | -0.28 |
| 220 | BG_L_6_5 | Tha_L_8_2 | SUB | SUB | -3.95 | -0.31 |
| 221 | Tha_L_8_5 | Tha_L_8_2 | SUB | SUB | -3.98 | -0.31 |
| 222 | Tha_L_8_6 | Tha_L_8_2 | SUB | SUB | -3.59 | -0.28 |
| 223 | Tha_L_8_6 | Tha_R_8_6 | SUB | SUB | -4.12 | -0.32 |
| 224 | Tha_L_8_6 | Tha_L_8_7 | SUB | SUB | -3.64 | -0.29 |
| 225 | Tha_L_8_6 | Tha_R_8_7 | SUB | SUB | -3.80 | -0.30 |

**Abbreviations:** DAN, Dorsal Attention Network; DMN, Default-Mode Network; FPN, Frontoparietal Network; SN, Salience Network; SMN, Sensory-Motor Network; SUB, Subcortex; VIS, Visual Network.

## S8. Developmental Stress and Task-Dependent Functional Connectivity

**Table S4.** Negative associations between prenatal stress and functional connectivity during emotion regulation (NBS-corrected).

| **Connection** | **Seed Region** | **Target Region** | **Seed Network** | **Target Network** | **t** | **Hedge's g** |
| --- | --- | --- | --- | --- | --- | --- |
| 1 | SFG_L_7_1 | SFG_R_7_1 | FPN | SN | -3.51 | -0.28 |
| 2 | MFG_L_7_5 | SFG_R_7_1 | DMN | SN | -3.57 | -0.28 |
| 3 | Tha_L_8_3 | SFG_L_7_5 | SUB | SMN | -3.56 | -0.28 |
| 4 | Tha_R_8_3 | SFG_L_7_5 | SUB | SMN | -3.58 | -0.28 |
| 5 | Tha_R_8_5 | SFG_L_7_5 | SUB | SMN | -4 | -0.32 |
| 6 | FuG_L_3_2 | SFG_R_7_5 | VIS | SMN | -3.63 | -0.29 |
| 7 | Tha_R_8_5 | SFG_R_7_5 | SUB | SMN | -4 | -0.32 |
| 8 | MFG_R_7_3 | MFG_L_7_1 | FPN | SN | -3.71 | -0.29 |
| 9 | IPL_R_6_2 | MFG_L_7_1 | FPN | SN | -3.59 | -0.28 |
| 10 | Tha_L_8_4 | MFG_L_7_1 | SUB | SN | -3.69 | -0.29 |
| 11 | Tha_L_8_7 | MFG_L_7_1 | SUB | SN | -3.53 | -0.28 |
| 12 | BG_R_6_5 | MFG_R_7_1 | SUB | FPN | -3.87 | -0.31 |
| 13 | Tha_R_8_7 | MFG_R_7_1 | SUB | FPN | -3.83 | -0.30 |
| 14 | OrG_L_6_3 | MFG_L_7_2 | Limbic | FPN | -3.86 | -0.30 |
| 15 | OrG_L_6_3 | MFG_R_7_2 | Limbic | FPN | -3.55 | -0.28 |
| 16 | BG_R_6_1 | MFG_L_7_4 | SUB | FPN | -3.55 | -0.28 |
| 17 | ITG_L_7_6 | MFG_R_7_4 | FPN | FPN | -3.50 | -0.28 |
| 18 | ITG_R_7_7 | MFG_R_7_4 | Limbic | FPN | -3.66 | -0.29 |
| 19 | Amyg_R_2_2 | MFG_R_7_4 | SUB | FPN | -3.63 | -0.29 |
| 20 | BG_L_6_1 | MFG_R_7_4 | SUB | FPN | -3.88 | -0.31 |
| 21 | BG_R_6_1 | MFG_R_7_4 | SUB | FPN | -3.57 | -0.28 |
| 22 | BG_R_6_5 | MFG_R_7_4 | SUB | FPN | -3.80 | -0.30 |
| 23 | Tha_L_8_7 | MFG_R_7_4 | SUB | FPN | -3.63 | -0.29 |
| 24 | BG_R_6_1 | MFG_R_7_5 | SUB | FPN | -3.89 | -0.31 |
| 25 | BG_R_6_5 | MFG_R_7_5 | SUB | FPN | -3.89 | -0.31 |
| 26 | STG_R_6_1 | IFG_L_6_1 | Limbic | FPN | -3.85 | -0.30 |
| 27 | SFG_L_7_7 | IFG_R_6_2 | DMN | FPN | -3.58 | -0.28 |
| 28 | MFG_R_7_5 | IFG_R_6_2 | FPN | FPN | -3.53 | -0.28 |
| 29 | Tha_R_8_6 | IFG_R_6_2 | SUB | FPN | -3.74 | -0.30 |
| 30 | Amyg_L_2_2 | IFG_L_6_3 | SUB | DMN | -3.62 | -0.29 |
| 31 | Tha_R_8_6 | IFG_R_6_4 | SUB | FPN | -3.54 | -0.28 |
| 32 | SFG_R_7_4 | IFG_L_6_5 | DAN | SN | -3.55 | -0.28 |
| 33 | Tha_L_8_6 | IFG_R_6_5 | SUB | SN | -3.58 | -0.28 |
| 34 | Amyg_R_2_2 | IFG_R_6_6 | SUB | SN | -3.67 | -0.29 |
| 35 | INS_L_6_5 | PrG_R_6_1 | SMN | SMN | -3.65 | -0.29 |
| 36 | Tha_R_8_5 | PrG_R_6_1 | SUB | SMN | -4.23 | -0.33 |
| 37 | Amyg_R_2_2 | PrG_L_6_3 | SUB | SMN | -3.50 | -0.28 |
| 38 | Hipp_L_2_2 | PrG_L_6_4 | SUB | SMN | -3.91 | -0.31 |
| 39 | Tha_R_8_5 | PrG_L_6_4 | SUB | SMN | -3.82 | -0.30 |
| 40 | ITG_R_7_2 | PrG_R_6_4 | DAN | SMN | -3.57 | -0.28 |
| 41 | FuG_L_3_2 | PrG_R_6_4 | VIS | SMN | -3.62 | -0.29 |
| 42 | MVOcC _L_5_4 | PrG_R_6_4 | VIS | SMN | -4.69 | -0.37 |
| 43 | MVOcC _R_5_4 | PrG_R_6_4 | VIS | SMN | -3.51 | -0.28 |
| 44 | Amyg_R_2_2 | PrG_R_6_4 | SUB | SMN | -3.89 | -0.31 |
| 45 | Hipp_L_2_2 | PrG_R_6_4 | SUB | SMN | -3.97 | -0.31 |
| 46 | SPL_R_5_1 | PrG_L_6_5 | DAN | SN | -3.73 | -0.29 |
| 47 | Tha_R_8_5 | PrG_L_6_5 | SUB | SN | -3.53 | -0.28 |
| 48 | INS_L_6_4 | PrG_R_6_5 | SN | SN | -3.61 | -0.29 |
| 49 | Tha_L_8_6 | PrG_R_6_5 | SUB | SN | -3.52 | -0.28 |
| 50 | Amyg_R_2_2 | PrG_R_6_6 | SUB | DAN | -3.60 | -0.28 |
| 51 | MVOcC _L_5_4 | PCL_L_2_1 | VIS | SN | -3.50 | -0.28 |
| 52 | MVOcC _L_5_4 | PCL_R_2_1 | VIS | SMN | -3.60 | -0.28 |
| 53 | MVOcC _L_5_4 | PCL_L_2_2 | VIS | SMN | -3.60 | -0.28 |
| 54 | MVOcC _L_5_4 | PCL_R_2_2 | VIS | SMN | -4.29 | -0.34 |
| 55 | INS_L_6_4 | STG_L_6_2 | SN | SMN | -3.79 | -0.30 |
| 56 | MFG_L_7_3 | STG_R_6_2 | FPN | SMN | -3.84 | -0.30 |
| 57 | MFG_R_7_3 | STG_R_6_2 | FPN | SMN | -3.93 | -0.31 |
| 58 | Tha_L_8_6 | STG_R_6_2 | SUB | SMN | -3.84 | -0.30 |
| 59 | Tha_L_8_6 | STG_R_6_3 | SUB | SMN | -3.56 | -0.28 |
| 60 | Tha_L_8_6 | STG_R_6_6 | SUB | DMN | -3.98 | -0.31 |
| 61 | Tha_R_8_6 | STG_R_6_6 | SUB | DMN | -3.90 | -0.31 |
| 62 | ITG_L_7_1 | ITG_R_7_1 | Limbic | Limbic | -4.25 | -0.34 |
| 63 | ITG_R_7_7 | ITG_R_7_1 | Limbic | Limbic | -4.40 | -0.35 |
| 64 | Tha_R_8_3 | ITG_R_7_1 | SUB | Limbic | -3.54 | -0.28 |
| 65 | Tha_R_8_5 | ITG_R_7_1 | SUB | Limbic | -3.61 | -0.29 |
| 66 | ITG_L_7_6 | ITG_L_7_3 | FPN | Limbic | -3.61 | -0.29 |
| 67 | ITG_R_7_7 | ITG_L_7_3 | Limbic | Limbic | -3.75 | -0.30 |
| 68 | Tha_R_8_5 | ITG_L_7_3 | SUB | Limbic | -3.97 | -0.31 |
| 69 | Tha_R_8_5 | ITG_R_7_3 | SUB | Limbic | -3.85 | -0.30 |
| 70 | ITG_R_7_7 | ITG_R_7_4 | Limbic | Limbic | -3.86 | -0.31 |
| 71 | BG_R_6_5 | ITG_R_7_5 | SUB | DAN | -3.81 | -0.30 |
| 72 | MFG_L_7_4 | FuG_L_3_2 | FPN | VIS | -3.71 | -0.29 |
| 73 | ITG_L_7_6 | PhG_R_6_1 | FPN | Limbic | -3.88 | -0.31 |
| 74 | Tha_R_8_6 | PhG_R_6_3 | SUB | VIS | -3.93 | -0.31 |
| 75 | BG_R_6_1 | SPL_L_5_3 | SUB | DAN | -3.90 | -0.31 |
| 76 | BG_R_6_1 | SPL_L_5_5 | SUB | DAN | -4.07 | -0.32 |
| 77 | BG_R_6_5 | SPL_L_5_5 | SUB | DAN | -3.50 | -0.28 |
| 78 | BG_R_6_1 | IPL_L_6_2 | SUB | FPN | -3.65 | -0.29 |
| 79 | Tha_L_8_7 | IPL_L_6_2 | SUB | FPN | -3.52 | -0.28 |
| 80 | BG_R_6_1 | IPL_R_6_3 | SUB | DAN | -3.90 | -0.31 |
| 81 | BG_R_6_5 | IPL_R_6_3 | SUB | DAN | -3.81 | -0.30 |
| 82 | BG_R_6_1 | IPL_L_6_4 | SUB | DMN | -3.63 | -0.29 |
| 83 | Tha_L_8_4 | IPL_L_6_4 | SUB | DMN | -3.66 | -0.29 |
| 84 | Tha_L_8_7 | IPL_L_6_4 | SUB | DMN | -4.02 | -0.32 |
| 85 | Tha_L_8_7 | IPL_R_6_4 | SUB | FPN | -3.69 | -0.29 |
| 86 | Tha_R_8_5 | IPL_L_6_6 | SUB | SMN | -3.78 | -0.30 |
| 87 | INS_L_6_4 | PoG_L_4_2 | SN | SMN | -3.74 | -0.30 |
| 88 | SFG_R_7_4 | PoG_L_4_4 | DAN | SMN | -4.02 | -0.32 |
| 89 | PrG_R_6_3 | PoG_L_4_4 | SMN | SMN | -4.61 | -0.36 |
| 90 | STG_L_6_5 | PoG_L_4_4 | Limbic | SMN | -4.28 | -0.34 |
| 91 | Amyg_R_2_2 | PoG_L_4_4 | SUB | SMN | -3.63 | -0.29 |
| 92 | Hipp_L_2_2 | PoG_L_4_4 | SUB | SMN | -3.54 | -0.28 |
| 93 | Tha_R_8_5 | PoG_L_4_4 | SUB | SMN | -4.58 | -0.36 |
| 94 | Tha_L_8_6 | PoG_L_4_4 | SUB | SMN | -3.84 | -0.30 |
| 95 | Tha_R_8_6 | PoG_L_4_4 | SUB | SMN | -3.55 | -0.28 |
| 96 | SFG_R_7_4 | PoG_R_4_4 | DAN | SMN | -4.01 | -0.32 |
| 97 | STG_L_6_3 | PoG_R_4_4 | SMN | SMN | -4.04 | -0.32 |
| 98 | INS_L_6_1 | PoG_R_4_4 | SMN | SMN | -3.66 | -0.29 |
| 99 | INS_R_6_4 | PoG_R_4_4 | SN | SMN | -4.02 | -0.32 |
| 100 | Amyg_R_2_2 | PoG_R_4_4 | SUB | SMN | -3.51 | -0.28 |
| 101 | BG_L_6_5 | PoG_R_4_4 | SUB | SMN | -3.63 | -0.29 |
| 102 | Tha_L_8_6 | PoG_R_4_4 | SUB | SMN | -3.65 | -0.29 |
| 103 | Tha_L_8_7 | PoG_R_4_4 | SUB | SMN | -4.79 | -0.38 |
| 104 | Tha_R_8_7 | PoG_R_4_4 | SUB | SMN | -3.57 | -0.28 |
| 105 | STG_R_6_1 | INS_L_6_1 | Limbic | SMN | -3.81 | -0.30 |
| 106 | PoG_R_4_1 | INS_L_6_1 | SMN | SMN | -3.75 | -0.30 |
| 107 | LOcC_L_4_4 | INS_L_6_1 | VIS | SMN | -3.56 | -0.28 |
| 108 | Amyg_L_2_2 | INS_R_6_1 | SUB | SMN | -3.56 | -0.28 |
| 109 | MFG_L_7_3 | INS_L_6_2 | FPN | SUB | -3.74 | -0.30 |
| 110 | MFG_L_7_4 | INS_L_6_2 | FPN | SUB | -3.78 | -0.30 |
| 111 | Tha_R_8_6 | INS_R_6_2 | SUB | FPN | -3.71 | -0.29 |
| 112 | SFG_R_7_4 | INS_L_6_3 | DAN | SN | -3.60 | -0.28 |
| 113 | Tha_L_8_6 | INS_R_6_3 | SUB | SN | -3.60 | -0.28 |
| 114 | ITG_L_7_1 | INS_R_6_6 | Limbic | SN | -3.66 | -0.29 |
| 115 | Tha_R_8_6 | CG_R_7_1 | SUB | DMN | -3.50 | -0.28 |
| 116 | Tha_L_8_7 | CG_R_7_1 | SUB | DMN | -3.51 | -0.28 |
| 117 | SFG_L_7_1 | CG_L_7_2 | FPN | SUB | -3.91 | -0.31 |
| 118 | SFG_L_7_2 | CG_L_7_2 | DMN | SUB | -4.84 | -0.38 |
| 119 | SFG_R_7_2 | CG_L_7_2 | FPN | SUB | -4.16 | -0.33 |
| 120 | SFG_L_7_3 | CG_L_7_2 | DMN | SUB | -4.66 | -0.37 |
| 121 | SFG_R_7_3 | CG_L_7_2 | DMN | SUB | -4.04 | -0.32 |
| 122 | SFG_R_7_4 | CG_L_7_2 | DAN | SUB | -3.69 | -0.29 |
| 123 | SFG_L_7_7 | CG_L_7_2 | DMN | SUB | -4.19 | -0.33 |
| 124 | SFG_R_7_7 | CG_L_7_2 | DMN | SUB | -3.61 | -0.29 |
| 125 | MFG_L_7_3 | CG_L_7_2 | FPN | SUB | -3.81 | -0.30 |
| 126 | MFG_R_7_3 | CG_L_7_2 | FPN | SUB | -4.95 | -0.39 |
| 127 | MFG_L_7_4 | CG_L_7_2 | FPN | SUB | -4.78 | -0.38 |
| 128 | MFG_R_7_4 | CG_L_7_2 | FPN | SUB | -4.28 | -0.34 |
| 129 | MFG_L_7_5 | CG_L_7_2 | DMN | SUB | -4.78 | -0.38 |
| 130 | MFG_R_7_5 | CG_L_7_2 | FPN | SUB | -4.12 | -0.33 |
| 131 | MFG_L_7_6 | CG_L_7_2 | DAN | SUB | -4.66 | -0.37 |
| 132 | IFG_R_6_5 | CG_L_7_2 | SN | SUB | -3.51 | -0.28 |
| 133 | OrG_R_6_2 | CG_L_7_2 | DMN | SUB | -4.15 | -0.33 |
| 134 | OrG_L_6_6 | CG_L_7_2 | DMN | SUB | -3.55 | -0.28 |
| 135 | OrG_R_6_6 | CG_L_7_2 | DMN | SUB | -3.67 | -0.29 |
| 136 | PrG_R_6_3 | CG_L_7_2 | SMN | SUB | -3.59 | -0.28 |
| 137 | IPL_L_6_2 | CG_L_7_2 | FPN | SUB | -4.65 | -0.37 |
| 138 | IPL_R_6_2 | CG_L_7_2 | FPN | SUB | -3.84 | -0.30 |
| 139 | IPL_L_6_4 | CG_L_7_2 | DMN | SUB | -4.47 | -0.35 |
| 140 | IPL_R_6_5 | CG_L_7_2 | DMN | SUB | -3.91 | -0.31 |
| 141 | PCun_L_4_3 | CG_L_7_2 | VIS | SUB | -3.74 | -0.30 |
| 142 | CG_L_7_1 | CG_L_7_2 | DMN | SUB | -3.57 | -0.28 |
| 143 | CG_R_7_1 | CG_L_7_2 | DMN | SUB | -3.97 | -0.31 |
| 144 | CG_L_7_3 | CG_L_7_2 | DMN | SUB | -4.44 | -0.35 |
| 145 | CG_L_7_4 | CG_L_7_2 | DMN | SUB | -3.68 | -0.29 |
| 146 | CG_R_7_4 | CG_L_7_2 | VIS | SUB | -3.68 | -0.29 |
| 147 | CG_L_7_7 | CG_L_7_2 | DMN | SUB | -4.01 | -0.32 |
| 148 | CG_R_7_7 | CG_L_7_2 | DMN | SUB | -4.45 | -0.35 |
| 149 | MVOcC _L_5_5 | CG_L_7_2 | VIS | SUB | -4.01 | -0.32 |
| 150 | Tha_L_8_6 | CG_R_7_3 | SUB | SN | -3.67 | -0.29 |
| 151 | MFG_R_7_3 | CG_L_7_5 | FPN | SN | -3.59 | -0.28 |
| 152 | MFG_L_7_4 | CG_L_7_5 | FPN | SN | -3.55 | -0.28 |
| 153 | CG_R_7_7 | CG_L_7_5 | DMN | SN | -3.65 | -0.29 |
| 154 | Tha_R_8_5 | CG_L_7_5 | SUB | SN | -3.56 | -0.28 |
| 155 | Tha_R_8_6 | CG_L_7_5 | SUB | SN | -3.98 | -0.31 |
| 156 | Tha_R_8_6 | CG_R_7_5 | SUB | SN | -3.55 | -0.28 |
| 157 | BG_R_6_5 | CG_L_7_6 | SUB | SN | -3.72 | -0.29 |
| 158 | Tha_L_8_7 | CG_L_7_6 | SUB | SN | -3.80 | -0.30 |
| 159 | Tha_R_8_7 | CG_L_7_6 | SUB | SN | -3.83 | -0.30 |
| 160 | BG_R_6_5 | CG_R_7_6 | SUB | SN | -4 | -0.32 |
| 161 | Tha_R_8_6 | CG_R_7_6 | SUB | SN | -3.64 | -0.29 |
| 162 | Tha_L_8_7 | CG_R_7_6 | SUB | SN | -3.83 | -0.30 |
| 163 | Tha_R_8_7 | CG_R_7_6 | SUB | SN | -3.86 | -0.31 |
| 164 | MFG_L_7_4 | CG_L_7_7 | FPN | DMN | -3.52 | -0.28 |
| 165 | IPL_R_6_2 | CG_L_7_7 | FPN | DMN | -3.77 | -0.30 |
| 166 | IPL_R_6_2 | CG_R_7_7 | FPN | DMN | -3.50 | -0.28 |
| 167 | Tha_R_8_6 | Amyg_R_2_1 | SUB | SUB | -3.58 | -0.28 |
| 168 | SFG_L_7_5 | Amyg_L_2_2 | SMN | SUB | -3.53 | -0.28 |
| 169 | Tha_L_8_1 | Amyg_L_2_2 | SUB | SUB | -4.10 | -0.32 |
| 170 | Tha_R_8_5 | Amyg_L_2_2 | SUB | SUB | -3.84 | -0.30 |
| 171 | Tha_L_8_6 | Amyg_L_2_2 | SUB | SUB | -3.84 | -0.30 |
| 172 | Tha_R_8_6 | Amyg_L_2_2 | SUB | SUB | -4.10 | -0.32 |
| 173 | Tha_L_8_7 | Amyg_L_2_2 | SUB | SUB | -3.87 | -0.31 |
| 174 | SPL_R_5_1 | Amyg_R_2_2 | DAN | SUB | -3.51 | -0.28 |
| 175 | Tha_R_8_6 | Hipp_L_2_2 | SUB | SUB | -3.88 | -0.31 |
| 176 | MFG_L_7_6 | BG_L_6_1 | DAN | SUB | -4.30 | -0.34 |
| 177 | IFG_R_6_2 | BG_L_6_1 | FPN | SUB | -4.20 | -0.33 |
| 178 | IFG_R_6_3 | BG_L_6_1 | DMN | SUB | -3.79 | -0.30 |
| 179 | Tha_R_8_5 | BG_L_6_1 | SUB | SUB | -3.71 | -0.29 |
| 180 | Tha_R_8_6 | BG_L_6_1 | SUB | SUB | -3.96 | -0.31 |
| 181 | MFG_L_7_4 | BG_R_6_1 | FPN | SUB | -3.88 | -0.31 |
| 182 | MFG_R_7_4 | BG_R_6_1 | FPN | SUB | -3.52 | -0.28 |
| 183 | MFG_L_7_6 | BG_R_6_1 | DAN | SUB | -3.74 | -0.30 |
| 184 | IFG_L_6_2 | BG_R_6_1 | FPN | SUB | -3.83 | -0.30 |
| 185 | IFG_R_6_2 | BG_R_6_1 | FPN | SUB | -4.31 | -0.34 |
| 186 | OrG_L_6_3 | BG_R_6_1 | Limbic | SUB | -3.59 | -0.28 |
| 187 | IFG_R_6_2 | BG_L_6_2 | FPN | SUB | -3.61 | -0.28 |
| 188 | Amyg_L_2_2 | BG_L_6_2 | SUB | SUB | -3.52 | -0.28 |
| 189 | Tha_L_8_6 | BG_L_6_2 | SUB | SUB | -3.75 | -0.30 |
| 190 | Tha_R_8_6 | BG_L_6_2 | SUB | SUB | -4.39 | -0.35 |
| 191 | IFG_L_6_2 | BG_R_6_2 | FPN | SUB | -3.69 | -0.29 |
| 192 | Tha_R_8_6 | BG_L_6_3 | SUB | SUB | -4.13 | -0.33 |
| 193 | IFG_R_6_2 | BG_R_6_3 | FPN | SUB | -3.52 | -0.28 |
| 194 | Tha_R_8_6 | BG_R_6_3 | SUB | SUB | -4.03 | -0.32 |
| 195 | IFG_L_6_2 | BG_L_6_4 | FPN | SUB | -3.72 | -0.29 |
| 196 | IFG_R_6_2 | BG_L_6_4 | FPN | SUB | -3.60 | -0.28 |
| 197 | IFG_L_6_3 | BG_L_6_4 | DMN | SUB | -3.64 | -0.29 |
| 198 | STG_R_6_4 | BG_L_6_4 | SMN | SUB | -3.76 | -0.30 |
| 199 | Tha_R_8_5 | BG_L_6_4 | SUB | SUB | -3.54 | -0.28 |
| 200 | Tha_R_8_6 | BG_L_6_4 | SUB | SUB | -5.06 | -0.40 |
| 201 | Tha_L_8_7 | BG_L_6_4 | SUB | SUB | -3.57 | -0.28 |
| 202 | CG_R_7_5 | BG_R_6_4 | SN | SUB | -3.52 | -0.28 |
| 203 | Tha_L_8_1 | BG_R_6_4 | SUB | SUB | -3.54 | -0.28 |
| 204 | Tha_R_8_6 | BG_R_6_4 | SUB | SUB | -3.70 | -0.29 |
| 205 | Tha_R_8_8 | BG_R_6_4 | SUB | SUB | -3.55 | -0.28 |
| 206 | MFG_L_7_3 | BG_L_6_5 | FPN | SUB | -3.71 | -0.29 |
| 207 | MFG_L_7_4 | BG_L_6_5 | FPN | SUB | -4.44 | -0.35 |
| 208 | IFG_L_6_3 | BG_L_6_5 | DMN | SUB | -3.57 | -0.28 |
| 209 | MFG_R_7_2 | BG_R_6_5 | FPN | SUB | -3.64 | -0.29 |
| 210 | MFG_L_7_3 | BG_R_6_5 | FPN | SUB | -3.68 | -0.29 |
| 211 | MFG_L_7_4 | BG_R_6_5 | FPN | SUB | -4.52 | -0.36 |
| 212 | MFG_R_7_4 | BG_R_6_5 | FPN | SUB | -3.73 | -0.30 |
| 213 | IFG_R_6_2 | BG_R_6_5 | FPN | SUB | -3.80 | -0.30 |
| 214 | OrG_L_6_3 | BG_R_6_5 | Limbic | SUB | -3.72 | -0.29 |
| 215 | Tha_L_8_1 | BG_L_6_6 | SUB | SUB | -4.01 | -0.32 |
| 216 | Tha_L_8_6 | BG_L_6_6 | SUB | SUB | -4.19 | -0.33 |
| 217 | Tha_R_8_6 | BG_L_6_6 | SUB | SUB | -4.71 | -0.37 |
| 218 | IFG_L_6_3 | BG_R_6_6 | DMN | SUB | -3.71 | -0.29 |
| 219 | IFG_R_6_3 | BG_R_6_6 | DMN | SUB | -3.72 | -0.29 |
| 220 | IFG_R_6_6 | BG_R_6_6 | SN | SUB | -4.06 | -0.32 |
| 221 | Tha_L_8_1 | BG_R_6_6 | SUB | SUB | -4.13 | -0.33 |
| 222 | Tha_L_8_6 | BG_R_6_6 | SUB | SUB | -3.96 | -0.31 |
| 223 | Tha_L_8_7 | BG_R_6_6 | SUB | SUB | -3.55 | -0.28 |
| 224 | Tha_R_8_8 | BG_R_6_6 | SUB | SUB | -3.75 | -0.30 |
| 225 | SFG_R_7_4 | Tha_L_8_1 | DAN | SUB | -3.93 | -0.31 |
| 226 | STG_R_6_1 | Tha_L_8_1 | Limbic | SUB | -4.06 | -0.32 |
| 227 | Amyg_L_2_2 | Tha_L_8_1 | SUB | SUB | -3.60 | -0.28 |
| 228 | MFG_L_7_3 | Tha_R_8_1 | FPN | SUB | -4 | -0.32 |
| 229 | MFG_R_7_3 | Tha_R_8_1 | FPN | SUB | -3.99 | -0.32 |
| 230 | MFG_L_7_4 | Tha_R_8_1 | FPN | SUB | -3.93 | -0.31 |
| 231 | MFG_R_7_4 | Tha_R_8_1 | FPN | SUB | -3.66 | -0.29 |
| 232 | MFG_L_7_5 | Tha_R_8_1 | DMN | SUB | -3.66 | -0.29 |
| 233 | MFG_R_7_5 | Tha_R_8_1 | FPN | SUB | -4.26 | -0.34 |
| 234 | MFG_R_7_7 | Tha_R_8_1 | FPN | SUB | -3.54 | -0.28 |
| 235 | OrG_R_6_2 | Tha_R_8_1 | DMN | SUB | -3.83 | -0.30 |
| 236 | OrG_L_6_6 | Tha_R_8_1 | DMN | SUB | -3.86 | -0.30 |
| 237 | OrG_R_6_6 | Tha_R_8_1 | DMN | SUB | -4.08 | -0.32 |
| 238 | IPL_R_6_4 | Tha_R_8_1 | FPN | SUB | -3.70 | -0.29 |
| 239 | IPL_R_6_5 | Tha_R_8_1 | DMN | SUB | -3.61 | -0.29 |
| 240 | Tha_R_8_6 | Tha_R_8_1 | SUB | SUB | -3.75 | -0.30 |
| 241 | Amyg_L_2_2 | Tha_L_8_2 | SUB | SUB | -3.53 | -0.28 |
| 242 | Tha_R_8_5 | Tha_L_8_2 | SUB | SUB | -3.87 | -0.31 |
| 243 | Tha_R_8_6 | Tha_L_8_2 | SUB | SUB | -4.81 | -0.38 |
| 244 | Tha_L_8_7 | Tha_L_8_2 | SUB | SUB | -3.98 | -0.31 |
| 245 | Tha_R_8_8 | Tha_L_8_2 | SUB | SUB | -4.20 | -0.33 |
| 246 | IFG_L_6_2 | Tha_R_8_2 | FPN | SUB | -3.75 | -0.30 |
| 247 | IFG_L_6_3 | Tha_R_8_2 | DMN | SUB | -3.63 | -0.29 |
| 248 | Tha_R_8_6 | Tha_L_8_3 | SUB | SUB | -3.56 | -0.28 |
| 249 | OrG_R_6_6 | Tha_R_8_3 | DMN | SUB | -3.60 | -0.28 |
| 250 | STG_L_6_2 | Tha_R_8_3 | SMN | SUB | -3.51 | -0.28 |
| 251 | OrG_L_6_3 | Tha_L_8_4 | Limbic | SUB | -3.51 | -0.28 |
| 252 | IFG_L_6_3 | Tha_R_8_4 | DMN | SUB | -3.55 | -0.28 |
| 253 | IFG_L_6_4 | Tha_R_8_4 | DMN | SUB | -3.53 | -0.28 |
| 254 | STG_L_6_3 | Tha_R_8_4 | SMN | SUB | -3.60 | -0.28 |
| 255 | SFG_R_7_4 | Tha_L_8_5 | DAN | SUB | -3.58 | -0.28 |
| 256 | OrG_R_6_6 | Tha_R_8_5 | DMN | SUB | -3.61 | -0.29 |
| 257 | MFG_R_7_5 | Tha_L_8_6 | FPN | SUB | -3.63 | -0.29 |
| 258 | IPL_R_6_2 | Tha_L_8_6 | FPN | SUB | -3.54 | -0.28 |
| 259 | MFG_R_7_5 | Tha_R_8_6 | FPN | SUB | -3.80 | -0.30 |
| 260 | MFG_L_7_3 | Tha_R_8_7 | FPN | SUB | -3.93 | -0.31 |
| 261 | MFG_R_7_3 | Tha_R_8_7 | FPN | SUB | -3.61 | -0.29 |
| 262 | MFG_L_7_4 | Tha_R_8_7 | FPN | SUB | -4.09 | -0.32 |
| 263 | MFG_R_7_4 | Tha_R_8_7 | FPN | SUB | -3.50 | -0.28 |
| 264 | MFG_R_7_5 | Tha_R_8_7 | FPN | SUB | -3.57 | -0.28 |
| 265 | OrG_L_6_3 | Tha_R_8_7 | Limbic | SUB | -3.59 | -0.28 |
| 266 | SFG_R_7_4 | Tha_L_8_8 | DAN | SUB | -3.63 | -0.29 |
| 267 | IFG_L_6_2 | Tha_L_8_8 | FPN | SUB | -3.54 | -0.28 |
| 268 | MFG_R_7_5 | Tha_R_8_8 | FPN | SUB | -4.04 | -0.32 |
| 269 | IFG_L_6_2 | Tha_R_8_8 | FPN | SUB | -3.74 | -0.30 |
| 270 | IFG_L_6_4 | Tha_R_8_8 | DMN | SUB | -3.53 | -0.28 |
| 271 | IFG_L_6_6 | Tha_R_8_8 | SN | SUB | -3.61 | -0.29 |
| 272 | OrG_R_6_2 | Tha_R_8_8 | DMN | SUB | -3.65 | -0.29 |
| 273 | OrG_L_6_6 | Tha_R_8_8 | DMN | SUB | -3.61 | -0.29 |
| 274 | OrG_R_6_6 | Tha_R_8_8 | DMN | SUB | -4.96 | -0.39 |
| 275 | PrG_R_6_3 | Tha_R_8_8 | SMN | SUB | -3.75 | -0.30 |
| 276 | STG_R_6_1 | Tha_R_8_8 | Limbic | SUB | -3.52 | -0.28 |
| 277 | IPL_R_6_5 | Tha_R_8_8 | DMN | SUB | -3.73 | -0.29 |
| 278 | PoG_R_4_4 | Tha_R_8_8 | SMN | SUB | -3.76 | -0.30 |
| 279 | CG_L_7_3 | Tha_R_8_8 | DMN | SUB | -3.50 | -0.28 |
| 280 | Tha_R_8_6 | Tha_R_8_8 | SUB | SUB | -3.87 | -0.31 |

**Abbreviations:** DAN, Dorsal Attention Network; DMN, Default-Mode Network; FPN, Frontoparietal Network; SN, Salience Network; SMN, Sensory-Motor Network; SUB, Subcortex; VIS, Visual Network.

**Table S5.** Top ten associations between prenatal stress exposure and functional connectivity during emotion regulation (based on t-values).

| Connection | Seed Region | Target Region | T-Value |
| --- | --- | --- | --- |
| 200 | Tha_R_8_6 | BG_L_6_4 | -5.06 |
| 274 | OrG_R_6_6 | Tha_R_8_8 | -4.96 |
| 126 | MFG_R_7_3 | CG_L_7_2 | -4.95 |
| 118 | SFG_L_7_2 | CG_L_7_2 | -4.84 |
| 243 | Tha_R_8_6 | Tha_L_8_2 | -4.81 |
| 103 | Tha_L_8_7 | PoG_R_4_4 | -4.79 |
| 127 | MFG_L_7_4 | CG_L_7_2 | -4.78 |
| 129 | MFG_L_7_5 | CG_L_7_2 | -4.78 |
| 217 | Tha_R_8_6 | BG_L_6_6 | -4.71 |
| 42 | MVOcC _L_5_4 | PrG_R_6_4 | -4.69 |


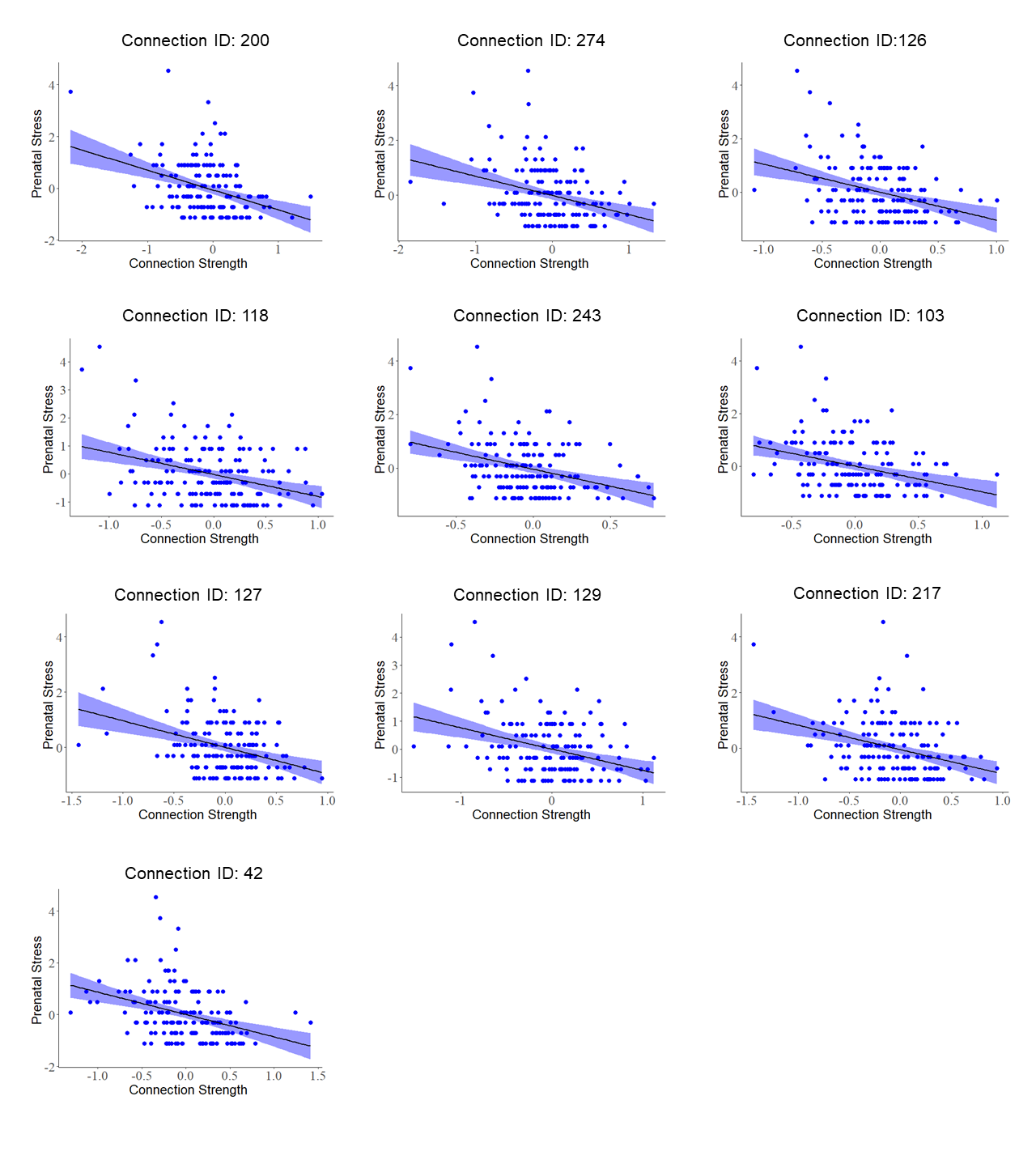


**Figure S6.** Scatter plots showing the associations between prenatal stress exposure and functional connectivity. Top ten associations based on t-values were visualized.

**Table S6.** Negative associations between childhood stress and functional connectivity during emotion regulation (NBS-corrected)

| **Connection** | **Seed Region** | **Target Region** | **Seed Network** | **Target Network** | **t** | **Hedge's g** |
| --- | --- | --- | --- | --- | --- | --- |
| 1 | OrG_L_6_1 | OrG_L_6_3 | DMN | LN | -3.65 | -0.18 |
| 2 | OrG_R_6_1 | OrG_L_6_3 | DMN | LN | -3.8 | -0.18 |
| 3 | Tha_R_8_8 | MTG_R_4_1 | SUB | FPN | -3.54 | -0.17 |
| 4 | Tha_R_8_1 | MTG_L_4_2 | SUB | DMN | -3.54 | -0.17 |
| 5 | Tha_R_8_8 | ITG_L_7_1 | SUB | LN | -3.64 | -0.17 |
| 6 | Tha_R_8_8 | ITG_L_7_2 | SUB | DAN | -3.58 | -0.17 |
| 7 | Tha_L_8_1 | ITG_L_7_4 | SUB | DMN | -3.79 | -0.18 |
| 8 | Tha_R_8_1 | ITG_L_7_4 | SUB | DMN | -4.2 | -0.2 |
| 9 | Tha_L_8_7 | ITG_L_7_4 | SUB | DMN | -3.69 | -0.18 |
| 10 | Tha_R_8_8 | ITG_L_7_4 | SUB | DMN | -3.98 | -0.19 |
| 11 | Tha_L_8_1 | ITG_R_7_4 | SUB | LN | -3.71 | -0.18 |
| 12 | Tha_R_8_8 | ITG_R_7_4 | SUB | LN | -3.72 | -0.18 |
| 13 | Tha_R_8_8 | ITG_L_7_6 | SUB | FPN | -3.76 | -0.18 |
| 14 | Tha_R_8_1 | ITG_R_7_6 | SUB | FPN | -3.51 | -0.17 |
| 15 | Tha_L_8_3 | ITG_R_7_6 | SUB | FPN | -4.31 | -0.21 |
| 16 | Tha_L_8_5 | ITG_R_7_6 | SUB | FPN | -4.03 | -0.19 |
| 17 | Tha_R_8_5 | ITG_R_7_6 | SUB | FPN | -3.5 | -0.17 |
| 18 | Tha_L_8_7 | ITG_R_7_6 | SUB | FPN | -3.73 | -0.18 |
| 19 | Tha_R_8_8 | ITG_R_7_6 | SUB | FPN | -4.13 | -0.2 |
| 20 | Tha_L_8_3 | ITG_L_7_7 | SUB | LN | -3.51 | -0.17 |
| 21 | Tha_R_8_5 | ITG_L_7_7 | SUB | LN | -3.95 | -0.19 |
| 22 | Tha_R_8_8 | ITG_L_7_7 | SUB | LN | -4.23 | -0.2 |
| 23 | Tha_L_8_1 | ITG_R_7_7 | SUB | LN | -4.09 | -0.2 |
| 24 | Tha_R_8_1 | ITG_R_7_7 | SUB | LN | -4.18 | -0.2 |
| 25 | Tha_L_8_3 | ITG_R_7_7 | SUB | LN | -4.1 | -0.2 |
| 26 | Tha_R_8_4 | ITG_R_7_7 | SUB | LN | -3.65 | -0.18 |
| 27 | Tha_L_8_5 | ITG_R_7_7 | SUB | LN | -3.99 | -0.19 |
| 28 | Tha_R_8_5 | ITG_R_7_7 | SUB | LN | -4.54 | -0.22 |
| 29 | Tha_L_8_7 | ITG_R_7_7 | SUB | LN | -4.17 | -0.2 |
| 30 | Tha_R_8_8 | ITG_R_7_7 | SUB | LN | -4.58 | -0.22 |
| 31 | SPL_R_5_2 | FuG_L_3_3 | DAN | DAN | -3.89 | -0.19 |
| 32 | STG_R_6_5 | pSTS_L_2_1 | LN | DMN | -3.59 | -0.17 |
| 33 | OrG_L_6_1 | SPL_R_5_2 | DMN | DAN | -3.98 | -0.19 |
| 34 | BG_R_6_1 | SPL_R_5_2 | SUB | DAN | -3.54 | -0.17 |
| 35 | OrG_L_6_1 | IPL_R_6_2 | DMN | FPN | -3.98 | -0.19 |
| 36 | OrG_L_6_1 | PCun_R_4_1 | DMN | FPN | -3.62 | -0.17 |
| 37 | SPL_R_5_2 | BG_R_6_1 | DAN | SUB | -4.08 | -0.2 |
| 38 | SPL_L_5_5 | BG_R_6_1 | DAN | SUB | -3.62 | -0.17 |
| 39 | BG_L_6_2 | BG_R_6_1 | SUB | SUB | -3.64 | -0.17 |
| 40 | STG_R_6_5 | Tha_L_8_7 | LN | SUB | -3.89 | -0.19 |
| 41 | SPL_R_5_2 | Tha_L_8_7 | DAN | SUB | -3.57 | -0.17 |
| 42 | BG_R_6_1 | Tha_R_8_8 | SUB | SUB | -3.69 | -0.18 |

**Abbreviations:** DAN, Dorsal Attention Network; DMN, Default-Mode Network; FPN, Frontoparietal Network; LH, left hemisphere; LN, Limbic Network; RH, right hemisphere; SN, Salience Network; SMN, Sensory-Motor Network; SUB, Subcortex; VIS, Visual Network.

**Table S7.** Top ten associations between childhood stress exposure and functional connectivity during emotion regulation (based-on t values).

| Connection | Seed Region | Target Region | T-Value |
| --- | --- | --- | --- |
| 30 | Tha_R_8_8 | ITG_R_7_7 | -4.58 |
| 28 | Tha_R_8_5 | ITG_R_7_7 | -4.54 |
| 15 | Tha_L_8_3 | ITG_R_7_6 | -4.31 |
| 22 | Tha_R_8_8 | ITG_L_7_7 | -4.23 |
| 8 | Tha_R_8_1 | ITG_L_7_4 | -4.20 |
| 24 | Tha_R_8_1 | ITG_R_7_7 | -4.18 |
| 29 | Tha_L_8_7 | ITG_R_7_7 | -4.17 |
| 19 | Tha_R_8_8 | ITG_R_7_6 | -4.13 |
| 25 | Tha_L_8_3 | ITG_R_7_7 | -4.10 |
| 23 | Tha_L_8_1 | ITG_R_7_7 | -4.09 |


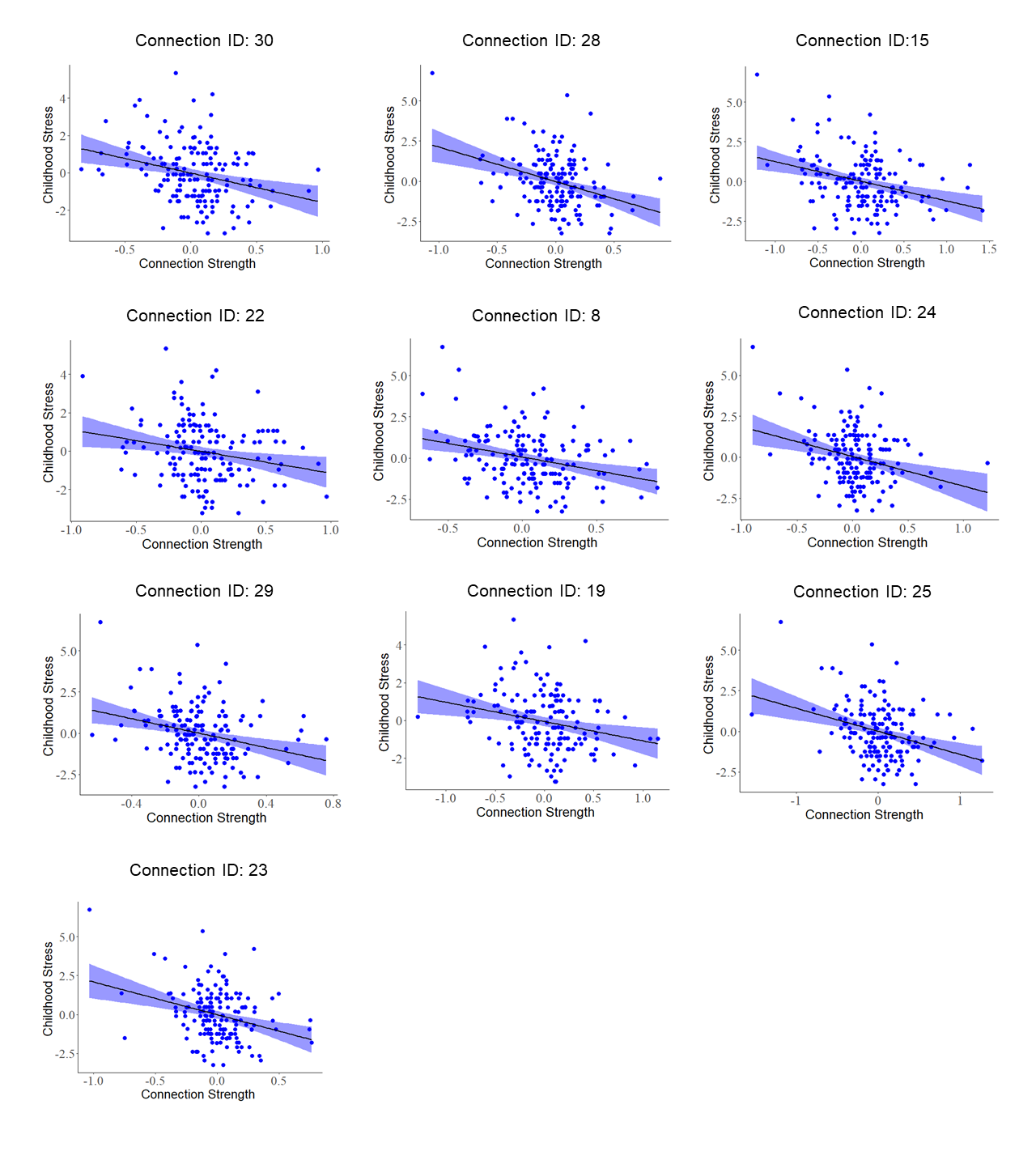


**Figure S7.** Scatter plots showing the associations between childhood stress exposure and functional connectivity. Top ten associations based on t-values were visualized.

**Table S8.** Negative associations between adolescence stress and functional connectivity during emotion regulation (NBS-corrected).

| **Connection** | **Seed Region** | **Target Region** | **Seed Network** | **Target Network** | **t** | **Hedge's g** |
| --- | --- | --- | --- | --- | --- | --- |
| 1 | CG_L_7_5 | SFG_R_7_3 | SN | DMN | 3.54 | 0.18 |
| 2 | CG_L_7_5 | SFG_L_7_7 | SN | DMN | 3.68 | 0.19 |
| 3 | INS_R_6_1 | SFG_R_7_7 | SMN | DMN | 3.95 | 0.21 |
| 4 | CG_L_7_2 | SFG_R_7_7 | SUB | DMN | 3.58 | 0.19 |
| 5 | CG_L_7_5 | SFG_R_7_7 | SN | DMN | 3.65 | 0.19 |
| 6 | CG_L_7_2 | MFG_R_7_7 | SUB | FPN | 3.53 | 0.18 |
| 7 | CG_L_7_5 | OrG_L_6_1 | SN | DMN | 3.77 | 0.20 |
| 8 | CG_L_7_5 | ITG_L_7_4 | SN | DMN | 4.04 | 0.21 |
| 9 | PCL_L_2_2 | ITG_L_7_7 | SMN | LN | 3.61 | 0.19 |
| 10 | INS_L_6_5 | ITG_L_7_7 | SMN | LN | 4.16 | 0.22 |
| 11 | CG_L_7_5 | ITG_L_7_7 | SN | LN | 4.29 | 0.22 |
| 12 | CG_L_7_5 | PhG_R_6_4 | SN | LN | 3.63 | 0.19 |
| 13 | CG_L_7_5 | CG_L_7_1 | SN | DMN | 3.72 | 0.19 |

**
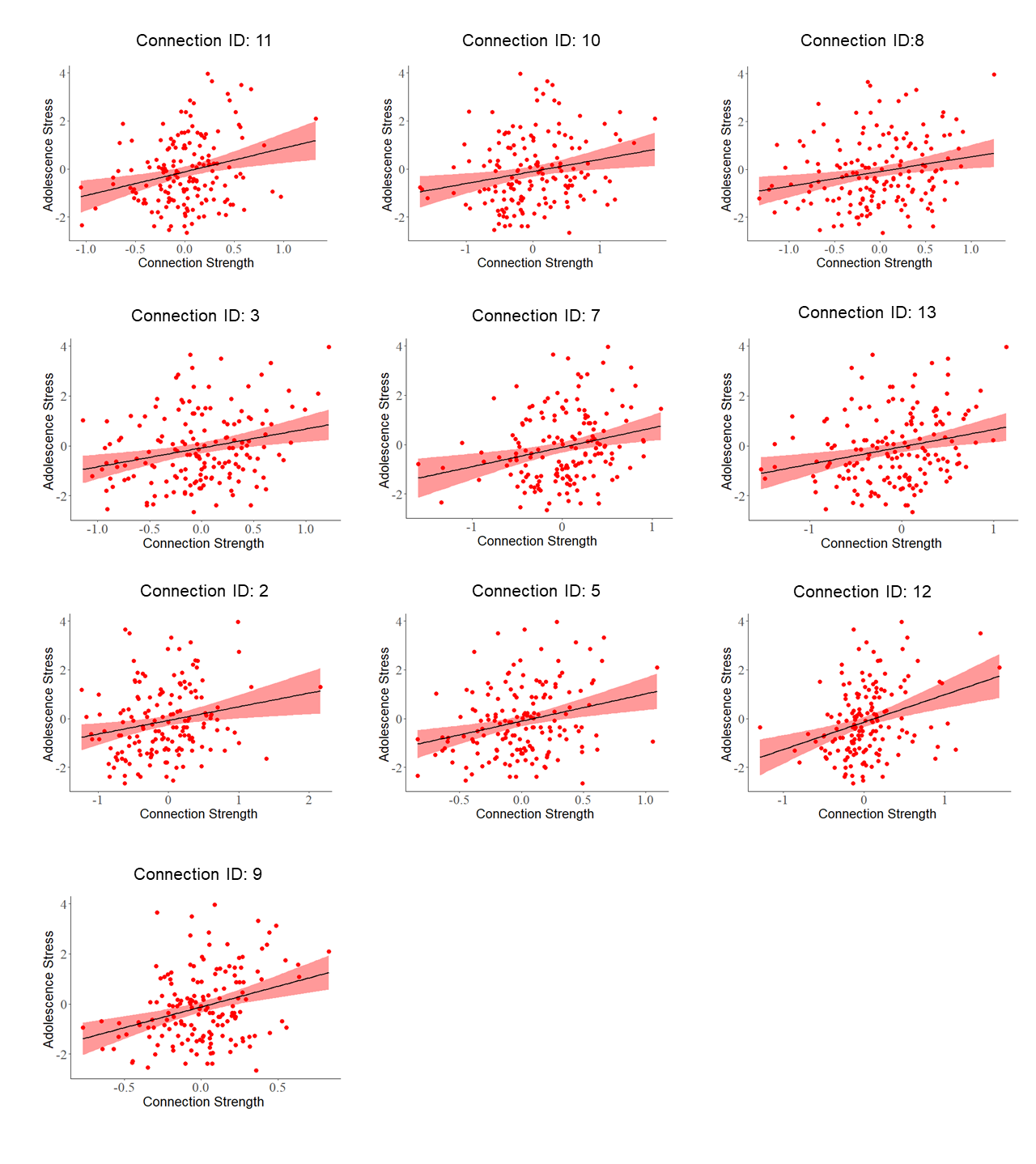
**

**Figure S8.** Scatter plots showing the associations between adolescence stress exposure and functional connectivity. Top ten associations based on t-values were visualized.

## S9. Brain-Behavior Relationship

**Table S9.** Mass univariate analysis (OLS) for connectivity parameters related to childhood stress and externalizing psychopathology relationship (uncorrected p < 0.05).

| Prenatal Stress | | | | | |
| --- | --- | --- | --- | --- | --- |
| Model 1: Base Model | | | | | |
| Symptom Dimension | Seed Region | Target Region | β | r2 | p |
| Internalizing | MVOcC _L_5_4 | PCL_L_2_1 | -3.63 | 0.03 | 0.022 |
|  | MVOcC _L_5_4 | PCL_R_2_1 | -4.38 | 0.03 | 0.012 |
|  | Tha_R_8_3 | ITG_R_7_1 | -4.18 | 0.04 | 0.006 |
|  | Tha_R_8_5 | ITG_R_7_1 | -5.41 | 0.04 | 0.005 |
|  | Tha_R_8_5 | ITG_L_7_3 | -5.05 | 0.03 | 0.014 |
|  | Tha_R_8_5 | ITG_R_7_3 | -5.64 | 0.04 | 0.01 |
|  | Tha_R_8_5 | PoG_L_4_4 | -3.74 | 0.02 | 0.032 |
|  | Amyg_R_2_2 | PoG_R_4_4 | -2.97 | 0.02 | 0.038 |
|  | IPL_R_6_2 | Tha_L_8_6 | -4.76 | 0.03 | 0.014 |
| Externalizing | Tha_R_8_5 | ITG_R_7_1 | -2.36 | 0.02 | 0.036 |
|  | Tha_R_8_5 | ITG_L_7_3 | -3.29 | 0.02 | 0.03 |
|  | Tha_R_8_5 | ITG_R_7_3 | -3.48 | 0.02 | 0.031 |
|  | Hipp_L_2_2 | PoG_L_4_4 | -1.72 | 0.02 | 0.036 |
|  | Tha_L_8_7 | BG_L_6_4 | 2.5 | 0.02 | 0.035 |
|  | OrG_R_6_6 | Tha_R_8_3 | -2.58 | 0.02 | 0.039 |
|  | IPL_R_6_2 | Tha_L_8_6 | -3.59 | 0.03 | 0.012 |
| Model 2: Controlled for other developmental periods | | | | | |
| Symptom Dimension | Seed Region | Target Region | β | r2 | p |
| Internalizing | IFG_R_6_3 | IPL_R_6_4 | 2.59 | 0.02 | 0.041 |
|  | CG_L_7_5 | CG_L_7_7 | -3.62 | 0.04 | 0.008 |
| Externalizing | Tha_R_8_6 | CG_L_7_6 | 2.16 | 0.02 | 0.037 |
|  | Tha_L_8_7 | BG_L_6_4 | 2.5 | 0.02 | 0.035 |
|  | Tha_L_8_7 | BG_L_6_6 | 3.11 | 0.02 | 0.029 |
| **Childhood Stress** | | | | | |
| Model 1: Base Model | | | | | |
| Symptom Dimension | Seed Region | Target Region | β | r2 | p |
| Internalizing | Tha_L_8_1 | ITG_L_7_4 | -4.63 | 0.02 | 0.043 |
| Model 2: Controlled for other developmental periods | | | | | |
| Symptom Dimension | Seed Region | Target Region | β | r2 | p |
| Internalizing | Tha_L_8_1 | ITG_L_7_4 | -4.63 | 0.02 | 0.043 |
| **Adolescence Stress** | | | | | |
| Model 2: Controlled for other developmental periods | | | | | |
| Symptom Dimension | Seed Region | Target Region | β | r2 | p |
| Internalizing | CG_R_7_6 | SFG_R_7_3 | -2.98 | 0.02 | 0.042 |
|  | CG_L_7_5 | OrG_R_6_1 | -3.11 | 0.02 | 0.028 |
|  | CG_R_7_5 | OrG_R_6_1 | -2.97 | 0.02 | 0.038 |
| Externalizing | CG_L_7_5 | MFG_R_7_6 | 2.19 | 0.02 | 0.04 |
|  | INS_R_6_3 | SPL_L_5_2 | -1.76 | 0.02 | 0.034 |

## S10. Sensitivity Analysis

### S10.1. Brain Parcellation

Whole-brain region-to-region connectivity requires a selection of a parcellation map, which could further introduce heterogeneity due to the subjective selection process (Hallquist & Hillary, 2018). To reduce this bias in our connectivity analyses, we repeated our analyses using two commonly used brain atlases in addition to Brainnetome atlas: Automated Anatomical Labelling (Rolls et al., 2020) representing anatomical parcellation and Schaefer atlas (Schaefer et al., 2018) representing functional parcellation with similar features (e.g., region number, network assignment). Since the Schafer atlas did not include subcortical regions, we combined it with the Melbourne subcortex atlas (Tian et al., 2020).

**Table S10.** Distribution of regional categories across the parcellation maps.

|  | AAL (n=164) | Brainnetome (n=246) | Schafer (n=232) |
| --- | --- | --- | --- |
| Frontal | 24 | 48 | 38 |
| Temporal | 14 | 54 | 31 |
| Occipital | 12 | 26 | 29 |
| Parietal | 10 | 27 | 35 |
| Sensory-Motor | 14 | 29 | 44 |
| Insula | 2 | 12 | 8 |
| Cingulum | 10 | 14 | 15 |
| Subcortex | 40  (28 Thalamus) | 36  (16 Thalamus) | 32  (4 Thalamus) |
| Midbrain | 12 | 0 | 0 |
| Cerebellum | 26 | 0 | 0 |

**Prenatal Stress**

Prenatal and newborn stress was negatively associated with functional connectivity in subcortical and frontal regions (Figure S9). In specific, outgoing connections from frontal regions to subcortex and outgoing connections from subcortex to other regions were affected. Subcortical connections included thalamus and striatum mostly. At network level, these alterations corresponded to outgoing connections from frontoparietal network (FPN), dorsal attention network (DAN), salience network (SN) and default-mode network (DMN) to subcortex and outgoing connections from subcortex to several networks (Figure S9). Similar results were found when the impact of stress occurring at other developmental periods was controlled (Figure S10).


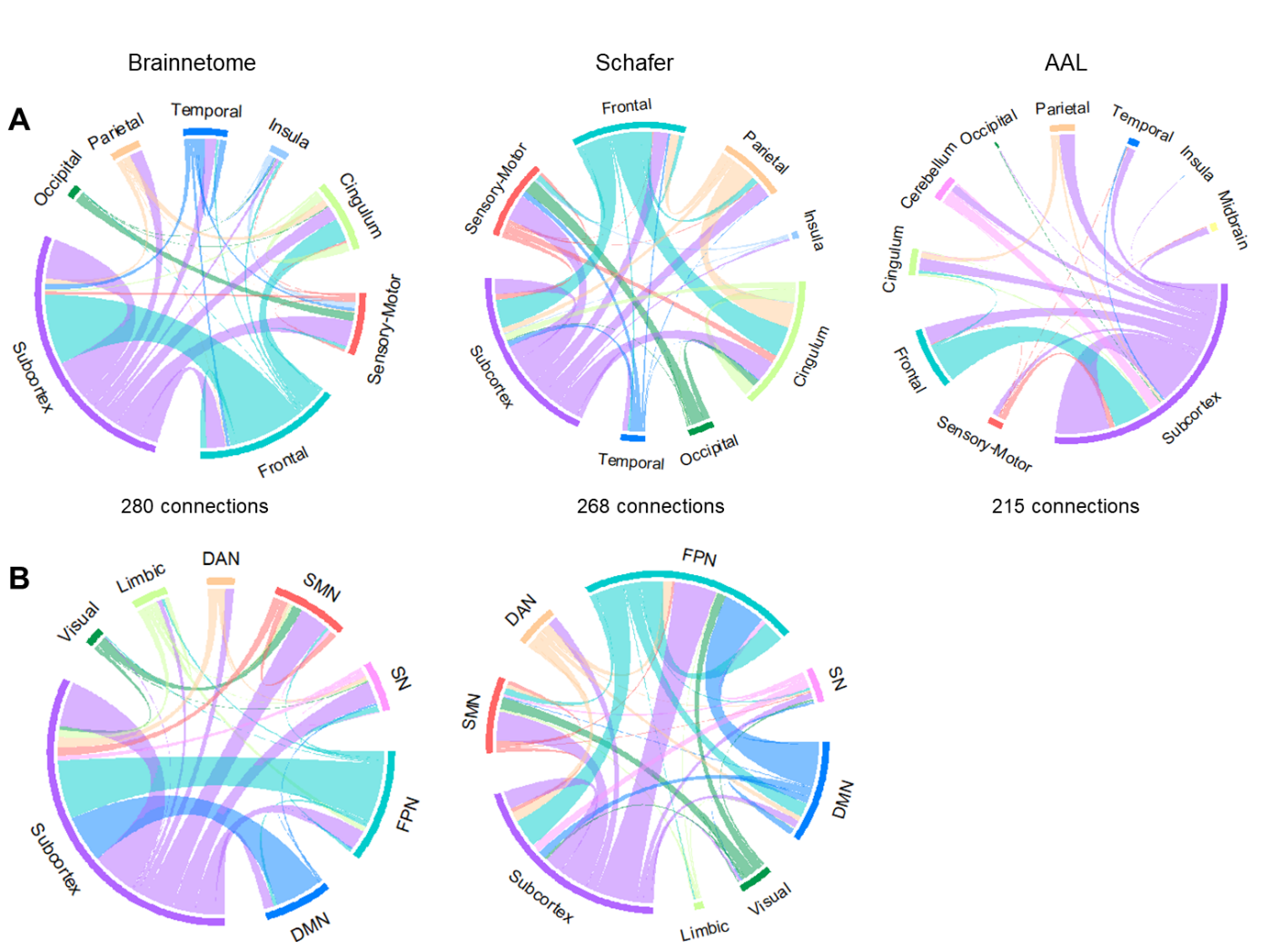
 **Figure S9.** Negative associations between prenatal stress and functional connectivity during emotion regulation at regional level (A) and network level (B). All results were corrected with NBS (corrected p < 0.001). Each region/network category is assigned to a specific color. Bundle color represents directionality. Connections arising from the source region are depicted with the color of the source region. Abbreviations: DAN, dorsal attention network; DMN, default-mode network; FPN, frontoparietal network; SMN, sensory-motor network; SN, salience network.


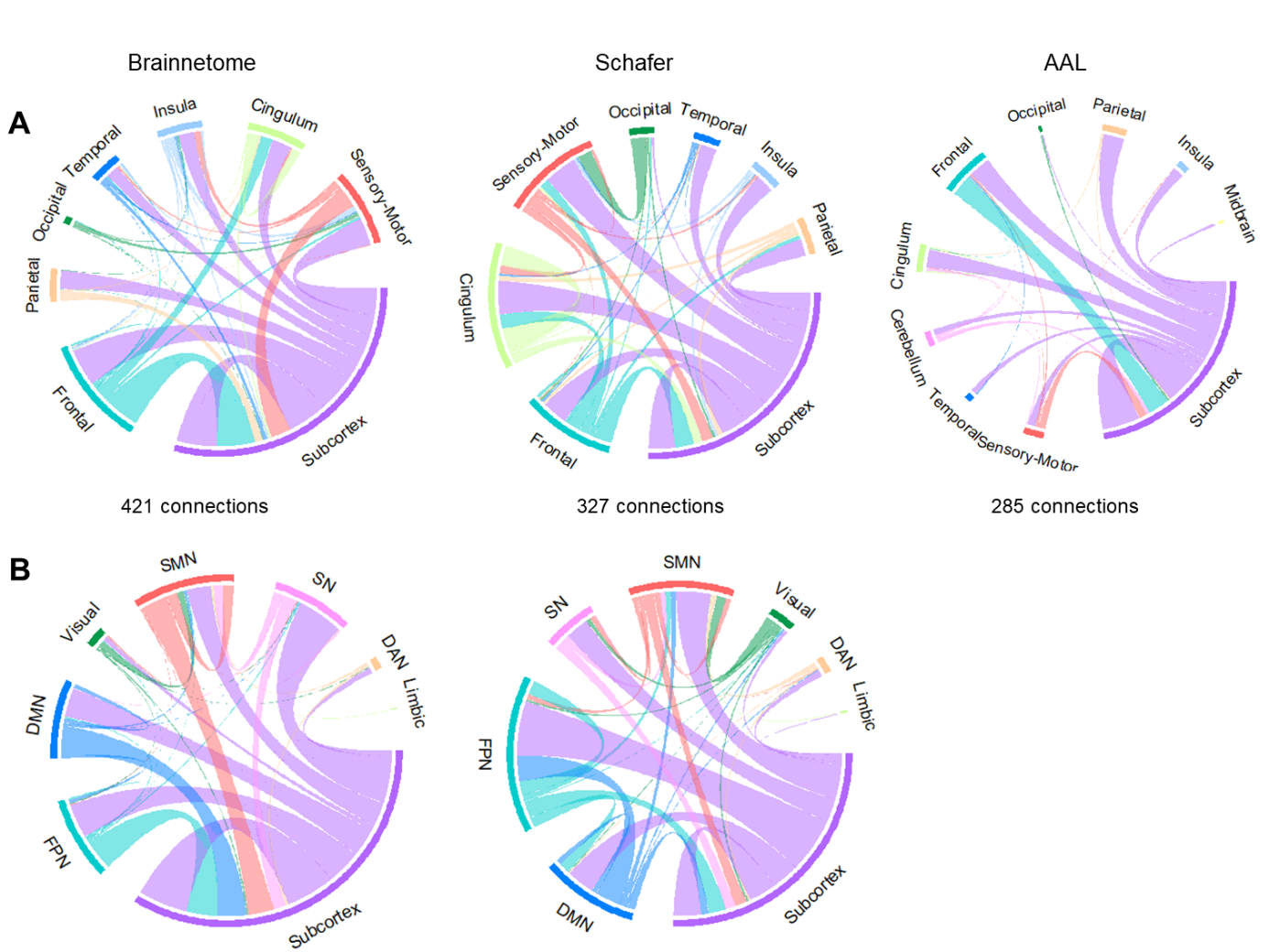


**Figure S10.** Negative associations between prenatal stress and functional connectivity during emotion regulation at regional level (A) and network level (B) **when the impact of other developmental periods was controlled**. All results were corrected with NBS (p < 0.001). Each region/network category is assigned to a specific color. Bundle color represents directionality. Connections arising from the source region are depicted with the color of the source region. Abbreviations: DAN, dorsal attention network; DMN, default-mode network; FPN, frontoparietal network; SMN, sensory-motor network; SN, salience network.

**Childhood Stress**

Childhood stress was negatively associated with functional connectivity in subcortical, temporal and parietal regions (Figure S11). Subcortical regions included mostly thalamus. These alterations corresponded to connections from subcortex to several networks including DMN and attention networks (FPN, DAN) (Figure S11). Similar alterations were present when the impact of other developmental periods was controlled. However, these changes were to lesser extent, mostly from subcortical regions to temporal regions corresponding the connections from subcortex to limbic network and DMN (Figure S12).


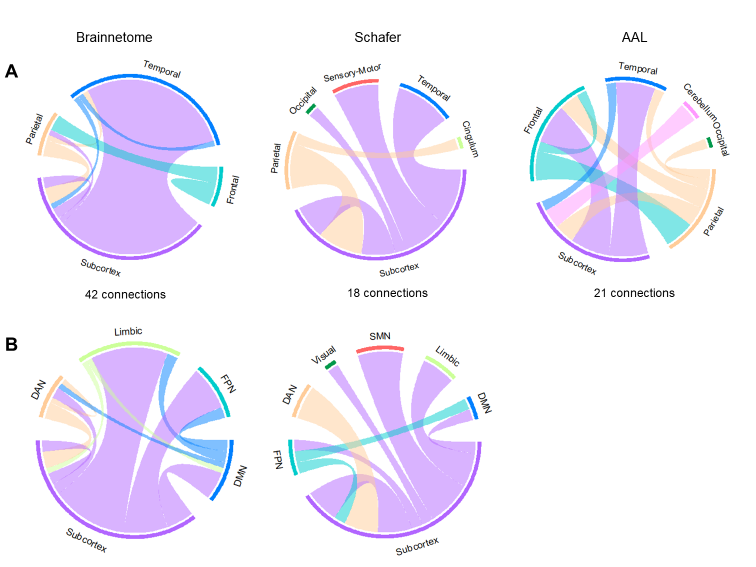


**Figure S11.** Negative associations between childhood stress and functional connectivity during emotion regulation at regional level (A) and network level (B). All results were corrected with NBS (p <0.05). Each region category is assigned to a specific color. Bundle color represents directionality. Connections arising from the source region are depicted with the color of the source region. Abbreviations: DAN, dorsal attention network; DMN, default-mode network; FPN, frontoparietal network; SMN, sensory-motor network; SN, salience network.


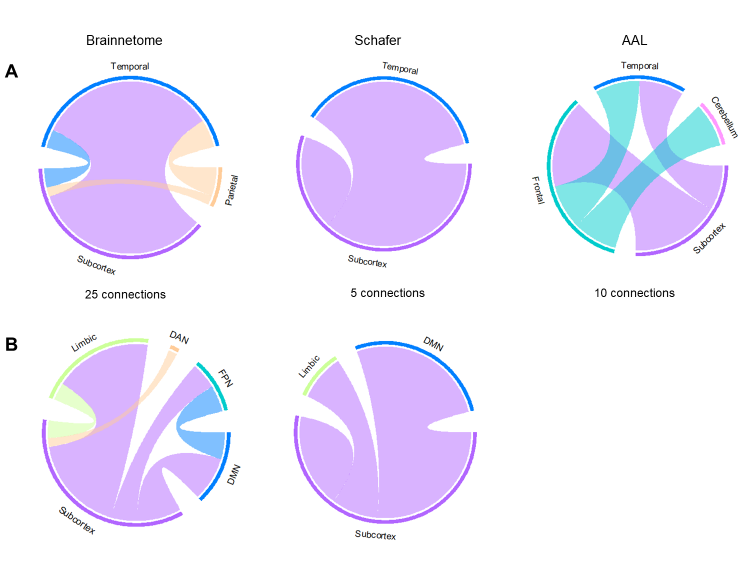


**Figure S12.** Negative associations between childhood stress and functional connectivity during emotion regulation at regional level (A) and network level (B) **when the impact of other developmental periods was controlled**. All results were corrected with NBS (corrected p < 0.05). Each region/network category is assigned to a specific color. Bundle color represents directionality. Connections arising from the source region are depicted with the color of the source region. Abbreviations: DAN, dorsal attention network; DMN, default-mode network; FPN, frontoparietal network.

**Adolescence Stress**

Only a few connections were related to adolescence stress positively. These changes were unique/not replicable across the atlases (Figure S13). We identified a large number of connectivity changes (n=114) when we controlled the impact of stress occurring at other developmental periods (Figure S14), which were replicable across the atlases.


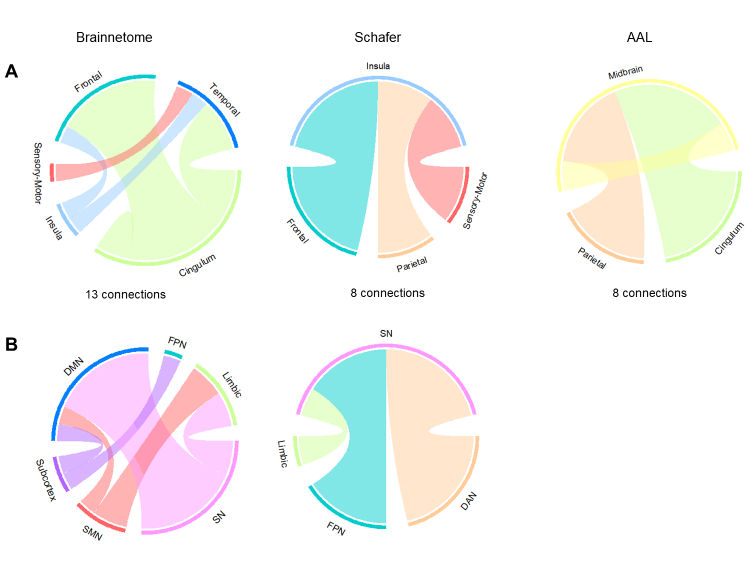


**Figure S13.** Positive associations between adolescence stress and functional connectivity during emotion regulation at regional level (A) and network level (B). All results were corrected with NBS (p <0.05). Each region category is assigned to a specific color. Bundle color represents directionality. Connections arising from the source region are depicted with the color of the source region. Abbreviations: DAN, dorsal attention network; DMN, default-mode network; FPN, frontoparietal network; SMN, sensory-motor network; SN, salience network.


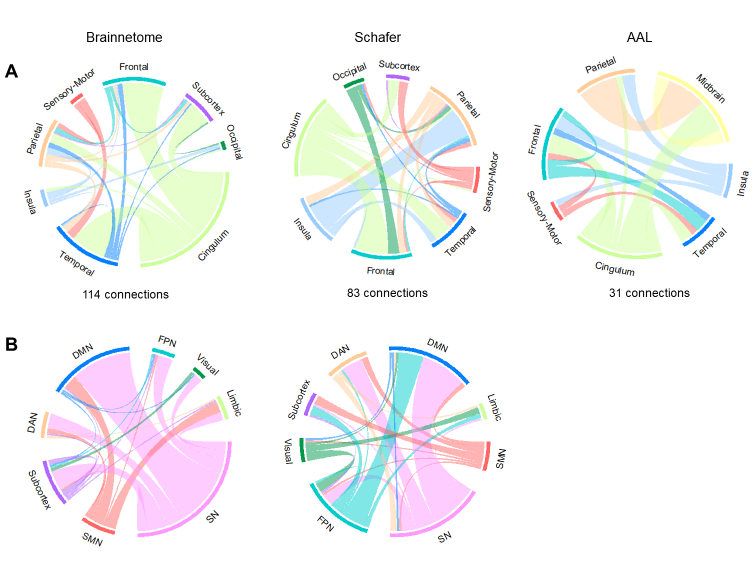


**Figure S14.** Positive associations between adolescence stress and functional connectivity during emotion regulation at regional level (A) and network level (B) **when the impact of other developmental periods was controlled**. All results were corrected with NBS (p < 0.001). Each region/network category is assigned to a specific color. Bundle color represents directionality. Connections arising from the source region are depicted with the color of the source region. Abbreviations: DAN, dorsal attention network; DMN, default-mode network; FPN, frontoparietal network; SMN, sensory-motor cortex; SN, salience network.

### S10.2. Impact of Self-Report

Parents reported the life events between T1 (3 months) and T5 (age 11). At T6 (age 15), both parent and youth reports were available, showing moderate correlations (r=0.49, p < 0.001). These measures differed in terms of item number with 50 in the parent version and 37 in the youth version. At T7 (age 19), only participants reported the life events. For the current study, we used the parent version for T6 and calculated total stress exposure for adolescence period by summing z-transformed T6 parent-report and T7 self-report scores. This decision was taken for compatibility reason, since the parent version contained roughly similar number of items with the T7 measure (n=53). However, since the adolescent stress scores reflected a summary of stress from different sources, we conducted additional analysis by combining T7 self-report scores with the T6 self –report scores. This measure (T6 self-report + T7 self-report) highly correlated with the previously reported adolescent stress scores (r=0.79, p <0.001).

As expected, results for adolescent stress based on self-reports were similar with the previously reported results. Higher adolescent stress was associated with increased connectivity from SN to cognitive networks, such as FPN, DAN and DMN (Figure S15).


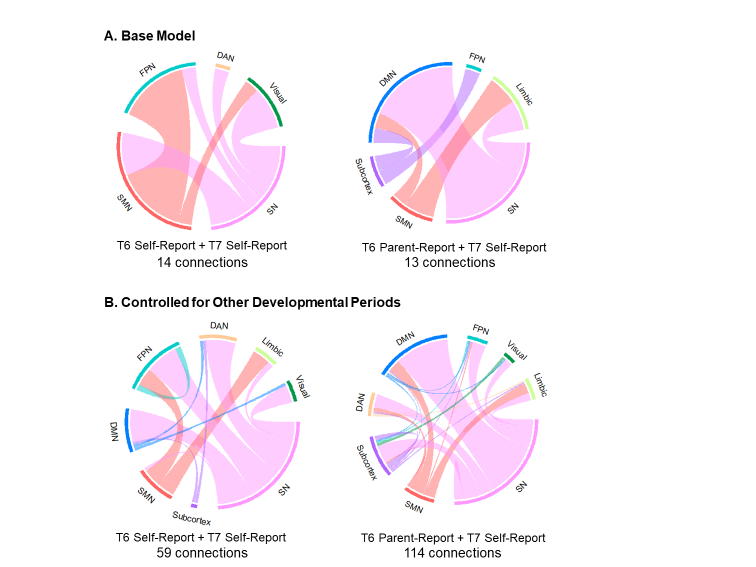


**Figure S15.** Connectivity changes related to adolescent stress. The scores were calculated using scores based on solely self-reported measures (left panel) versus the reports from mixed sources (right panel). All results were corrected with NBS (corrected p < 0.001). Each network category is assigned to a specific color. Bundle color represents directionality. Connections arising from the source region are depicted with the color of the source region. Abbreviations: DAN, dorsal attention network; DMN, default-mode network; FPN, frontoparietal network; SMN, sensory-motor network; SN, salience network.

### S10.3. Regulation Strategy

**
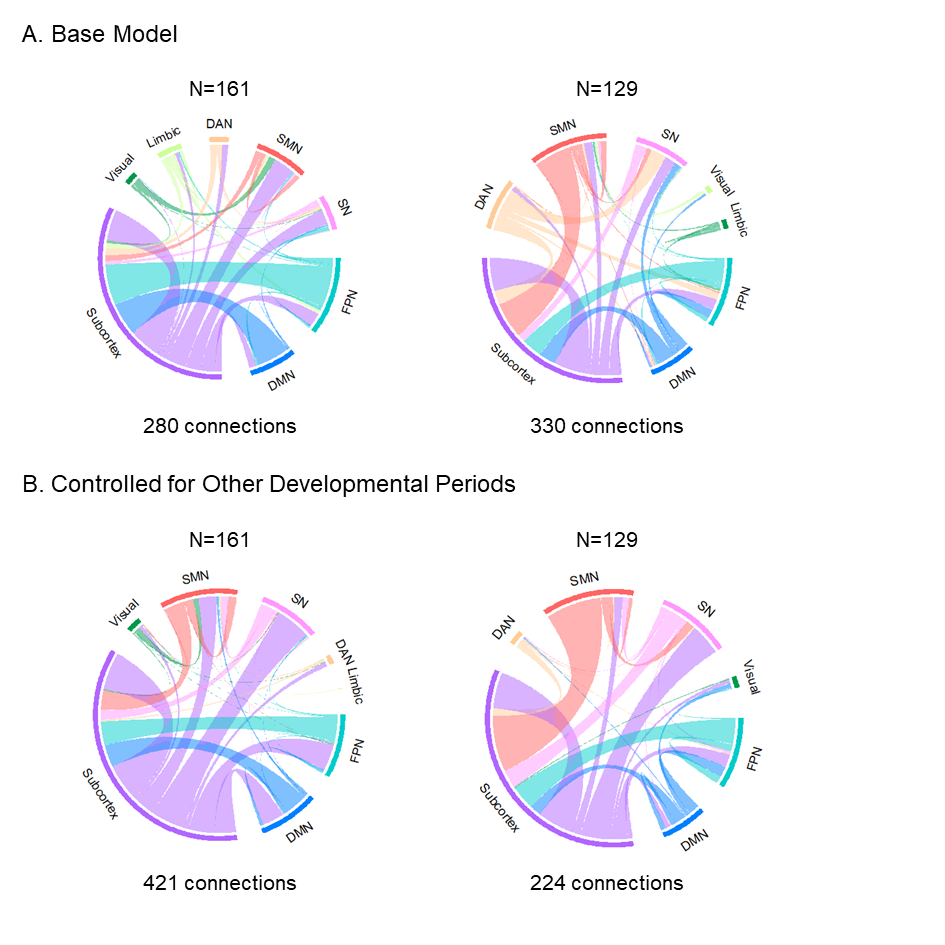
Figure S16.** Connectivity changes related to prenatal stress in full sample (left) and when the participants who did not use reappraisal were excluded (right). All results were corrected with NBS (corrected p < 0.001). Each network category is assigned to a specific color. Bundle color represents directionality. Connections arising from the source region are depicted with the color of the source region. Abbreviations: DAN, dorsal attention network; DMN, default-mode network; FPN, frontoparietal network; SMN, sensory-motor network; SN, salience network.

**
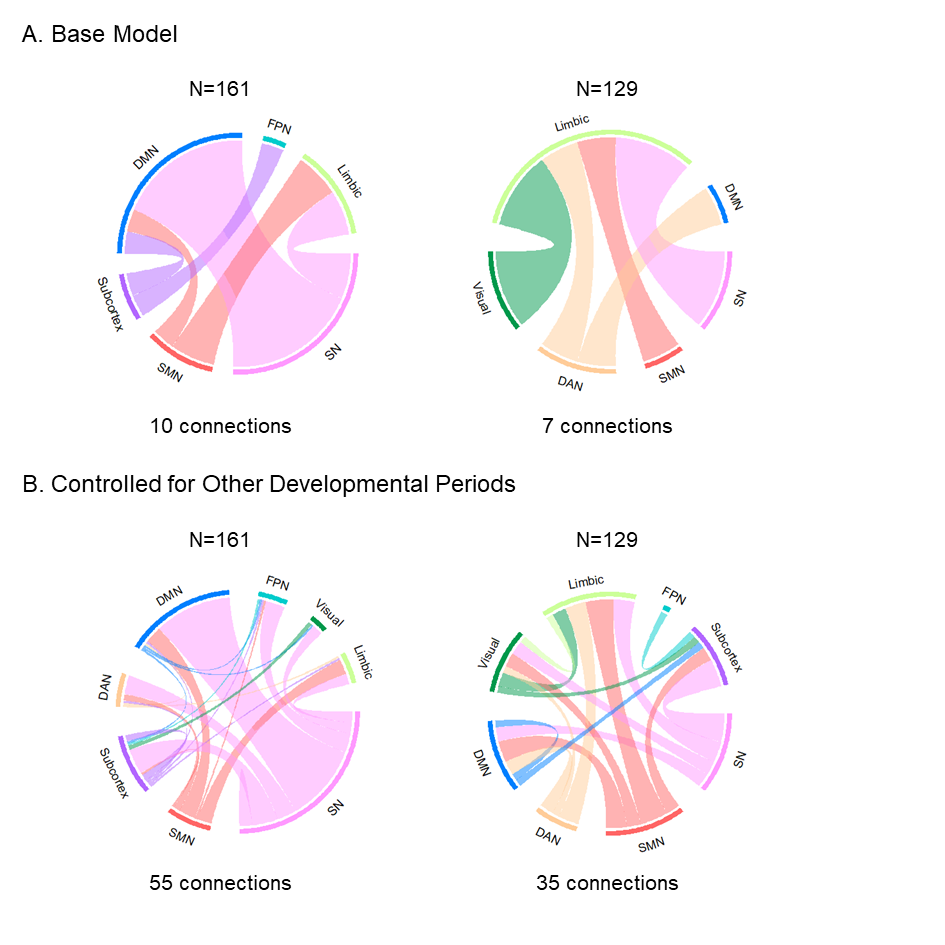
**

**Figure S17.** Connectivity changes related to adolescence stress in full sample (left) and when the participants who did not use reappraisal were excluded (right). All results were corrected with NBS (corrected p < 0.001). Each network category is assigned to a specific color. Bundle color represents directionality. Connections arising from the source region are depicted with the color of the source region. Abbreviations: DAN, dorsal attention network; DMN, default-mode network; FPN, frontoparietal network; SMN, sensory-motor network; SN, salience network.

**References**

Achenbach, T. M. (1991). *Integrative Guide for the 1991 CBCL/4-18, YSR, and TRF Profiles*. University of Vermont Department of Psychiatry.

Achenbach, T. M. (1997). *Manual for the Young Adult Self-Report and Yound Adult Behavior Checklist*. University of Vermont Department of Psychiatry.

Achenbach, T. M., & Rescorla, L. A. (2003). *Manual for the ASEBA adult forms & profiles. Research Center for Children, Youth, & Families*.

Aldao, A., Nolen-Hoeksema, S., & Schweizer, S. (2010). Emotion-regulation strategies across psychopathology: A meta-analytic review. In *Clinical Psychology Review* (Vol. 30, Issue 2). https://doi.org/10.1016/j.cpr.2009.11.004

Gerchen, M. F., Kirsch, P., & Feld, G. B. (2021). Brain-wide inferiority and equivalence tests in fMRI group analyses: Selected applications. *Human Brain Mapping*, *42*(18). https://doi.org/10.1002/hbm.25664

Gross, J. J., & John, O. P. (2003). Individual Differences in Two Emotion Regulation Processes: Implications for Affect, Relationships, and Well-Being. *Journal of Personality and Social Psychology*, *85*(2). https://doi.org/10.1037/0022-3514.85.2.348

Hallquist, M. N., & Hillary, F. G. (2018). Graph theory approaches to functional network organization in brain disorders: A critique for a brave new small-world. *Network Neuroscience*, *3*(1). https://doi.org/10.1162/netn_a_00054

Laucht, M., Esser, G., Baving, L., Gerhold, M., Hoesch, I., Ihle, W., Steigleider, P., Stock, B., Stoehr, R. M., Weindrich, D., & Schmidt, M. H. (2000). Behavioral sequelae of perinatal insults and early family adversity at 8 years of age. *Journal of the American Academy of Child and Adolescent Psychiatry*, *39*(10), 1229–1237. https://doi.org/10.1097/00004583-200010000-00009

Maier-Diewald, W., Wittchen, H.-U., Hecht, H., & Werner-Eilert, K. (1983). *Die Münchner Ereignisliste (MEL) - Anwendungsmanual*.

Marquardt, D. W., & Snee, R. D. (1975). Ridge regression in practice. *American Statistician*, *29*(1). https://doi.org/10.1080/00031305.1975.10479105

Rolls, E. T., Huang, C. C., Lin, C. P., Feng, J., & Joliot, M. (2020). Automated anatomical labelling atlas 3. *NeuroImage*, *206*. https://doi.org/10.1016/j.neuroimage.2019.116189

Schaefer, A., Kong, R., Gordon, E. M., Laumann, T. O., Zuo, X.-N., Holmes, A. J., Eickhoff, S. B., & Yeo, B. T. T. (2018). Local-Global Parcellation of the Human Cerebral Cortex from Intrinsic Functional Connectivity MRI. *Cerebral Cortex*, *28*(9). https://doi.org/10.1093/cercor/bhx179

Tian, Y., Margulies, D. S., Breakspear, M., & Zalesky, A. (2020). Topographic organization of the human subcortex unveiled with functional connectivity gradients. *Nature Neuroscience*, *23*(November). http://dx.doi.org/10.1038/s41593-020-00711-6

Tibshirani, R. (1996). Regression Shrinkage and Selection Via the Lasso. *Journal of the Royal Statistical Society: Series B (Methodological)*, *58*(1). https://doi.org/10.1111/j.2517-6161.1996.tb02080.x

Ying, X. (2019). An Overview of Overfitting and its Solutions. *Journal of Physics: Conference Series*, *1168*(2). https://doi.org/10.1088/1742-6596/1168/2/022022

Zalesky, A., Fornito, A., & Bullmore, E. T. (2010). Network-based statistic: Identifying differences in brain networks. *NeuroImage*, *53*(4). https://doi.org/10.1016/j.neuroimage.2010.06.041
